# Epigenetic Patterns in Blood Associated With Lipid Traits Predict Incident Coronary Heart Disease Events and Are Enriched for Results From Genome-Wide Association Studies

**DOI:** 10.1161/CIRCGENETICS.116.001487

**Published:** 2017-02-20

**Authors:** Åsa K. Hedman, Michael M. Mendelson, Riccardo E. Marioni, Stefan Gustafsson, Roby Joehanes, Marguerite R. Irvin, Degui Zhi, Johanna K. Sandling, Chen Yao, Chunyu Liu, Liming Liang, Tianxiao Huan, Allan F. McRae, Serkalem Demissie, Sonia Shah, John M. Starr, L. Adrienne Cupples, Panos Deloukas, Timothy D. Spector, Johan Sundström, Ronald M. Krauss, Donna K. Arnett, Ian J. Deary, Lars Lind, Daniel Levy, Erik Ingelsson

**Affiliations:** From the Department of Medical Sciences, Molecular Epidemiology and Science for Life Laboratory (Å.K.H., S.G., E.I.) and Department of Medical Sciences, Molecular Medicine and Science for Life Laboratory (J.K.S.), Uppsala University, Sweden; Cardiovascular Medicine unit, Department of Medicine Solna, Karolinska Institute, Stockholm, Sweden (Å.K.H.) Framingham Heart Study, MA (M.M.M., R.J., C.Y., C.L., T.H., S.D., L.A.C., D.L.); Department of Biostatistics (C.L., L.A.C., S.D.), Boston University, MA; Boston University, MA (M.M.M.); Department of Cardiology, Boston Children’s Hospital, MA (M.M.M.); Population Sciences Branch, National Heart, Lung, and Blood Institute, National Institutes of Health, Bethesda, MD (M.M.M., R.J., C.Y., C.L., T.H., D.L.); Centre for Cognitive Ageing and Cognitive Epidemiology (R.E.M., J.M.S., I.J.D.), Medical Genetics Section, Centre for Genomics and Experimental Medicine, Institute of Genetics and Molecular Medicine (R.E.M.), Alzheimer Scotland Dementia Research Centre (J.M.S.), and Department of Psychology (I.J.D.), University of Edinburgh, United Kingdom; Queensland Brain Institute, The University of Queensland, Brisbane, Australia (R.E.M., A.F.M., S.S.); Institute for Molecular Bioscience, University of Queensland, Brisbane, Queensland, Australia (S.S., A.F.M.); Hebrew Senior Life, Harvard Medical School, Boston, MA (R.J.); Department of Epidemiology, School of Public Health (M.R.I.) and Department of Biostatistics, Section on Statistical Genetics (D.Z.), University of Alabama at Birmingham; Department of Biostatistics, Harvard School of Public Health, Boston, MA (L. Liang); William Harvey Research Institute, Barts and The London School of Medicine and Dentistry, Queen Mary University of London, United Kingdom (P.D.); Princess Al-Jawhara Al-Brahim Centre of Excellence in Research of Hereditary Disorders (PACER-HD), King Abdulaziz University, Jeddah, Saudi Arabia (P.D.); Department of Twin Research and Genetic Epidemiology, King’s College London, United Kingdom (T.D.S.); Deparment of Medical Sciences, Cardiovascular Epidemiology, Uppsala University Hospital, Sweden (J.S., L.L.); Children’s Hospital Oakland Research Institute, CA (R.M.K.); College of Public Health, University of Kentucky, Lexington (D.K.A.); and Department of Medicine, Division of Cardiovascular Medicine, Stanford University School of Medicine, CA (E.I.).

**Keywords:** cardiovascular diseases, DNA Methylation, epigenomics, gene expression, lipids

## Abstract

**Background—:**

Genome-wide association studies have identified loci influencing circulating lipid concentrations in humans; further information on novel contributing genes, pathways, and biology may be gained through studies of epigenetic modifications.

**Methods and Results—:**

To identify epigenetic changes associated with lipid concentrations, we assayed genome-wide DNA methylation at cytosine–guanine dinucleotides (CpGs) in whole blood from 2306 individuals from 2 population-based cohorts, with replication of findings in 2025 additional individuals. We identified 193 CpGs associated with lipid levels in the discovery stage (*P*<1.08E-07) and replicated 33 (at Bonferroni-corrected *P*<0.05), including 25 novel CpGs not previously associated with lipids. Genes at lipid-associated CpGs were enriched in lipid and amino acid metabolism processes. A differentially methylated locus associated with triglycerides and high-density lipoprotein cholesterol (HDL-C; cg27243685; *P*=8.1E-26 and 9.3E-19) was associated with *cis*-expression of a reverse cholesterol transporter (*ABCG1; P*=7.2E-28) and incident cardiovascular disease events (hazard ratio per SD increment, 1.38; 95% confidence interval, 1.15–1.66; *P*=0.0007). We found significant *cis*-methylation quantitative trait loci at 64% of the 193 CpGs with an enrichment of signals from genome-wide association studies of lipid levels (*P*_TC_=0.004, *P*_HDL-C_=0.008 and *P*_triglycerides_=0.00003) and coronary heart disease (*P*=0.0007). For example, genome-wide significant variants associated with low-density lipoprotein cholesterol and coronary heart disease at *APOB* were *cis*-methylation quantitative trait loci for a low-density lipoprotein cholesterol–related differentially methylated locus.

**Conclusions—:**

We report novel associations of DNA methylation with lipid levels, describe epigenetic mechanisms related to previous genome-wide association studies discoveries, and provide evidence implicating epigenetic regulation of reverse cholesterol transport in blood in relation to occurrence of cardiovascular disease events.

## Abstract 2

Supplemental Digital Content is available in the text.

Cardiovascular disease (CVD) is the leading cause of death worldwide.^1^ Serum concentrations of total cholesterol (TC) and subcomponents of low-density lipoprotein cholesterol (LDL-C), high-density lipoprotein cholesterol (HDL-C), and triglycerides are established risk factors for coronary heart disease (CHD).^1^ Recent studies have provided evidence of causal roles for LDL-C and triglycerides in CHD.^2,3^ Further understanding of the genomic regulatory mechanisms linking lipids to CHD may enhance our ability to predict CHD risk, tailor current CHD treatments, or discover new treatments for CHD.

**See Clinical Perspective**

Genome-wide association studies (GWAS) have been successful in identifying numerous single-nucleotide polymorphisms (SNPs) associated with lipid levels and CHD.^4,5^ Because many of the SNPs are located in noncoding regions, epigenetic mechanisms can be suspected to mediate many of the genetic discoveries. Integrative analyses of methylation of cytosine nucleotides at cytosine–guanine dinucleotide (CpG) sites with genetic sequence variants and gene expression may elucidate previously unknown genes and pathways underlying GWAS discoveries. In addition to variation in DNA methylation that is determined by the surrounding genetic sequence,^6^ methylation is also affected by early exposures in utero^7,8^ and later life environmental factors.^9,10^ Environmentally induced alterations in DNA methylation may mediate environmental contributions to disease^11^ and reveal novel genes and pathways involved in disease that cannot be discovered in GWAS alone. Regulation of gene expression via DNA methylation may explain an additional component of interindividual variation in lipid levels beyond genetic sequence variants. Because much of the population burden of dyslipidemia and CHD is not explained by GWAS loci, relating differential DNA methylation to gene expression, intermediate metabolites, and disease end points may be useful in identifying additional candidate genes and mechanisms for which directed perturbation may help prevent morbidity and mortality from CHD.

In this study, we aimed to identify epigenetic variation in relation to lipid levels through epigenome-wide association analyses of whole blood–derived DNA in ≤2306 individuals with independent external replication of findings in ≤2025 individuals. Methylation differences in blood-derived DNA have been shown to reflect transtissue differential methylation in various tissues,^12–14^ including liver^15^ and adipose.^16^ In addition to the discovery of lipid-related differential DNA methylation, we assessed the association of lipid-related epigenetic changes to the risk of incident CHD events. Finally, we combined lipid-associated DNA methylation with genetic sequence variants, gene expression, and intermediate metabolites in an attempt to unravel the underlying genomic regulatory mechanisms linking serum lipid measures to CHD risk.

## Methods

### Study Participants and Design

**Figure 1.**
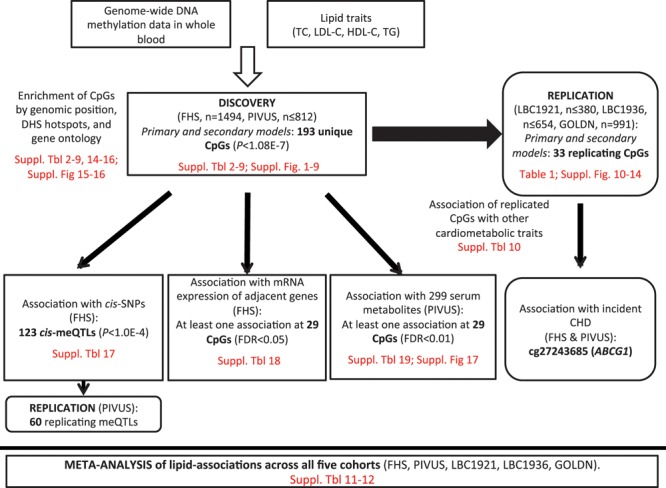
Overview of the study. CpG indicates cytosine–guanine dinucleotide; FDR, false discovery rate; FHS, Framingham Heart Study; GOLDN, Genetics of Lipid Lowering Drugs and Diet Network; HDL-C, high-density lipoprotein cholesterol; LBC, Lothian Birth Cohorts; LDL-C, low-density lipoprotein cholesterol; meQTL, methylation quantitative trait locus; PIVUS, Prospective Investigation of the Vasculature in Uppsala Seniors Study; TC, total cholesterol; and TG, triglyceride.

We conducted an epigenome-wide association study of serum lipid concentrations (TC, HDL-C, LDL-C, and triglycerides) in over 4000 adult participants from large community-based cohorts in the United States and Europe (Figure 1). Ethical approvals for the project were granted by the local Ethics Committee for each of the participating cohorts, and all samples were collected after obtaining written and signed informed consent. Participants from the FHS (Framingham Heart Study) offspring cohort (n=1494; mean [SD] age=66.4 [8.9] years)^17^ and the PIVUS (Prospective Investigation of the Vasculature in Uppsala Seniors Study; n≤812; 70.2 [0.2] years)^18^ were included in the discovery analysis. Loci identified as significant in the discovery (*P*<1.08E-07; Bonferroni-adjusted *P* value for multiple testing) were then examined for external replication in participants from the LBC1921 (Lothian Birth Cohorts of 1921; n≤380; 79.1 [0.6] years) and LBC1936 (LBC of 1936; n≤654; 69.5 [0.8] years)^19–21^ and the GOLDN (Genetics of Lipid Lowering Drugs and Diet Network; n=991; 48.8 [16] years).^22^ Characteristics of the cohorts are available in Table I in the Data Supplement. Further details about cohort-specific study design and sample collection are available in Methods in the Data Supplement. Primary analyses examined the association of each lipid component with methylation levels in blood at 459 433 CpGs and were adjusted for age, sex, white cell counts (if applicable), and batch effects; secondary models additionally adjusted for body mass index (BMI). We excluded individuals taking lipid medications (statins, fibrates, etc.) because the cross-sectional design would not allow us to determine if DNA methylation changes contributed to elevated lipids necessitating lipid medications or were secondary to medication use. The identified differentially methylation loci were assessed for associations with nearby genetic sequence variants in *cis* (defined as ±100 kb), intermediate phenotypes (gene expression and metabolites in blood), and incident CHD events.

### Phenotype Measurements and Disease Outcomes

Lipids traits were measured in blood samples collected after fasting with the exception of LBC (LBC1921 and LBC1936) for which nonfasting blood was drawn. Lipid measurements were performed using standard methods as described in Methods in the Data Supplement for each study. In FHS, PIVUS, and LBC1936, LDL-C levels were calculated by the Friedewald equation, whereas levels were directly measured in GOLDN. In LBC1921, HDL-C and LDL-C were not available. Characteristics of the lipid traits for each cohort are available in Table I in the Data Supplement. Weight and height were measured in each study using standardized protocols. BMI was calculated as weight in kg divided by height in m^2^. In FHS and PIVUS, cardiovascular events during ≤10 years of follow-up (adjudicated by physicians) were used to define a composite CHD end point, which included fatal or nonfatal myocardial infarction and revascularization procedure (percutaneous transluminal coronary angioplasty or coronary artery bypass graft). In FHS, data on coronary death and coronary insufficiency (unstable angina) were also included.

### Genome-Wide DNA Methylation Profiling

Genome-wide DNA methylation profiling was performed on genomic DNA isolated from whole blood (FHS, PIVUS, LBC1921, and LBC1936) or CD4^+^ T cells (GOLDN). DNA samples were bisulphite converted and analyzed on Illumina HumanMethylation450 BeadChip (Illumina Inc, San Diego, CA) following the manufactures’ protocol. After quality control procedures, methylation data were available for analyses in 2377 FHS, 967 PIVUS, 446 LBC1921, 920 LBC1936, and 995 GOLDN participants. Further cohort-specific details and quality control procedures are available in Methods in the Data Supplement. In all studies, blood used in extraction of DNA for methylation analysis was collected at the same time point as phenotype and covariate measurements.

### Additional Molecular Genomics Data

In FHS, SNP data were obtained from the Affymetrix 550K Array (Affymetrix, Santa Clara, CA) and imputed to 1000 Genomes SNPs (phase 1 release), as previously reported.^23^ The FHS genotype data are available at Database of Genotypes and Phenotypes under the accession number phs000342.v13.p9. In PIVUS, individuals were genotyped using the Illumina OmniExpress and Illumina Metabochip microarrays. Data were imputed to 1000G (version: March 2012) using Impute v.2.2.2.^24^ Gene expression profiles in blood, obtained using the Affymetrix Human Exon 1.0 ST GeneChip platform, were available for 2246 participants in the FHS. Untargeted metabolomic profiles in serum were available for 785 PIVUS participants also included in the lipid-association analyses. Acquity Ultra Performance Liquid Chromatography coupled to a Xevo G2 Q-TOFMS (Waters Corporation, Milford, MA) was used in metabolomic profiling. Only annotated metabolites (n=229) were used in analysis in relation to DNA methylation. Further details are available in Methods in the Data Supplement.

### Annotation of DNA Methylation Probes

Mapping and annotation of the 485 764 probes on the HumanMethylation450K BeadChip have previously been described.^25^ Only autosomal probes were included in analyses. Briefly, probes mapping to multiple locations (with at least 2 mismatches) in the human reference genome (GRCh37) were excluded. Furthermore, probes were filtered based on SNPs as follows: those with a common SNP (minor allele frequency>5%) within 10 bp of the methylation site and those overlapping copy number variants were excluded from analysis. This resulted in a final set of probes which were assigned to CpG islands and RefSeq transcripts downloaded from the UCSC Genome Browser. Probes within 2 kb away from borders of a CpG island were defined as shores and those within 2 kb of shores as falling within shelves. The rest were assigned to others/open sea. Probes were mapped in relation to transcripts as follows: TSS1500 (1500–200 bp upstream of transcription start site), TSS200 (200 bp upstream of transcription start site), the 5′-UTR (untranslated region), the first exon, the gene body, or the 3′-UTR.^26^

### Statistical Analysis

#### Association of Methylation of Blood Cell–Derived DNA With Lipids

Multivariable linear regression models were conducted (using cohort-specific approaches described in Methods in the Data Supplement) with DNA methylation β value specified as the dependent variable and the lipid component as the independent variable of interest. The primary model was adjusted for age, sex, white cell count (if applicable), technical covariates, and, if applicable, family structure (included as random effects using the R packages *pedigreemm*^27^ [FHS] or *kinship*^28^ [GOLDN], see further details in Methods in the Data Supplement). Secondary models additionally adjusted for BMI. Individuals on lipid-lowering medications were excluded from all analyses. Lipid levels (in mg/dL) were analyzed on the raw scale, except levels of triglyceride that were natural log-transformed before analyses. Probes with a common SNP (minor allele frequency>5%) within 10 bp of the methylation site were excluded from analysis. Fixed-effect meta-analyses were performed using the inverse variance–weighted method implemented in METAL^29^ of genome-wide association results in the discovery cohorts (FHS and PIVUS). CpGs significant at Bonferroni-corrected α threshold <0.05 (taking the number of CpGs into account; corresponding to a nominal *P*<1.08E-7) in discovery were analyzed in the replication cohorts. Meta-analyses of the results in the individual replication cohorts (LBC1921, LBC1936, and GOLDN) were performed using the same method as above.

#### Cross-Tissue Validation of Lipid Associations

Lipid-associated CpGs in blood were validated in DNA methylation data from subcutaneous abdominal adipose tissue (SAT) from the MuTHER (Multiple Tissue Human Expression Resource) study.^30^ This study and data set is described in detail in Grundberg et al.^25^ The study contains genome-wide DNA methylation data using the Illumina HumanMethylation450 array collected from 648 female twins and singletons (97 monozygotic pairs, 162 dizygotic pairs, and 130 singletons) of European ancestry. The participants had a mean age of ≈60 years and a mean BMI of 26.6 kg/m^2^. After removing individuals on lipid-lowering medication and with missing phenotype, a total of 588, 588, 589, and 639 participants were considered in the analyses of TC, LDL-C, HDL-C, and triglycerides, respectively. For association with phenotype, a linear mixed effects model was fitted which was adjusted for age, bisulphite conversion concentration, bisulphite conversion efficiency, and BeadChip as fixed effects and family relationship (twin pairing) and zygosity as random effects. One-hundred sixty-four out of 193 lipid-associated CpGs could be tested in SAT.

#### Gene Set Enrichment Analysis

To place our data in the context of biological processes or pathways, we subjected genes annotated to CpG sites (from 1500 bp upstream of transcription start site to 3′-UTR)^26^ associated with phenotypes to pathway analysis using Database for Annotation, Visualization and Integrated Discovery (DAVID).^31,32^ We used annotations from the Kyoto Encyclopedia of Genes and Genomes, Protein Analysis Through Evolutionary Relationships, Gene Ontology, REACTOME, and Clusters of Orthologous Groups of proteins.

#### Methylation Quantitative Trait Locus Analysis

Methylation quantitative trait locus (meQTL) analysis for lipid-associated methylation probes was performed in the FHS cohort (n=2246), and significant lead meQTL SNPs (*P*<1E-04) were tested for replication in the PIVUS cohort (n=775). MeQTL analysis was limited to SNPs located within 100 kb either side of the probe location (*cis*) and SNPs with a minor allele frequency >5% and imputation quality Rsq >0.8. In FHS, the residual of the DNA methylation β value was extracted after the removal of the fixed (age, sex, and imputed white cell counts using the Houseman method^33^) and random covariates (chip, row, and column), along with the kinship correlation structure. The DNA methylation residual was regressed on the SNP genotype additionally adjusting for 25 methylation principal components to account for unmeasured technical variation. Imputed SNPs were entered into the model as allele dosages. In PIVUS, the association between normalized methylation β values and posterior mean genotypes (MACH format) was modeled by a linear mixed effect model, using R^34^ and the *lmer* function (lme4 package), fitted by maximum likelihood assuming a normally distributed error term. Models were adjusted for age, sex, and predicted white cell counts (estimated from the DNA methylation data using the Houseman algorithm^33^ as implemented in R package *minfi*^35^) as fixed effects and chip, chip row, and chip column as random effects.

#### Association With Gene Expression Data

In FHS, the association between DNA methylation and gene expression (available in 2246 participants with DNA methylation) was performed on the gene expression residuals after the removal of the fixed and random covariates, along with the kinship correlation structure using a linear model, primarily to avoid potential confounding by blood count. Only CpGs that were methylome-wide significant were tested, and individual CpGs were tested against a single gene expression transcript in the regression model. All gene transcripts within ±500 kb (cis) of the CpG were assessed.

#### Association With Targeted Metabolites

In PIVUS, the associations between normalized methylation β values at lipid-associated CpGs and 229 serum metabolites were modeled by a linear mixed effect model, using R^34^ and the *lmer* function (lme4 package), fitted by maximum likelihood assuming a normally distributed error term. Models were adjusted for age, sex, and predicted white cell counts (using the Houseman algorithm^33^ in R package *minfi*^35^) as fixed effects and chip, chip row, and chip column as random effects. False discovery rate (FDR) were estimated based on *Q* values.^36^

#### Association With Disease Outcome

In FHS, Cox models were fitted in R using the *coxme* package to model the association of baseline DNA methylation with incident CHD events adjusted for age, sex (fixed effects), and family structure (mixed effect) for the 33 replicated lipid-associated CpGs. As using measured technical covariates (chip, row, and column) with a binary outcome resulted in too many overall levels, surrogate variable analysis (that capture sources of heterogeneity in the methylation data and can be used to control for the influence of these latent variables on inference)^37^ was used to capture the measured and unmeasured technical variation in the methylation data, and 5 surrogate variables (associated with incident CHD at *P* value <0.05) were included as covariates in the model.

In PIVUS, Cox models were fitted in R using the *coxph* function in the survival package, to model the association between case/control status and standardized methylation levels at the 33 replicated lipid-associated CpGs. Models were adjusted for age, sex, chip, and predicted white cell counts (using the Houseman algorithm^33^ in the R package *minfi*^35^).

## Results

### Associations of DNA Methylation With Lipid Levels in Blood

We sought to examine whether differences in DNA methylation were associated with circulating lipid levels (study design and main results outlined in Figure 1). After meta-analysis of 459 433 CpGs in the FHS (n=1494) and PIVUS (n=812) studies, we found methylation at 40, 23, 110, and 28 CpG sites associated with TC, LDL-C, HDL-C, and triglycerides, respectively, at methylome-wide significant threshold (*P*<1.08E-7; Volcano plots in Figures I through IV in the Data Supplement; Manhattan plots in Figures V through VIII in the Data Supplement). In total, there were 184 unique CpG sites (annotated to 138 unique genes) associated with any lipid level (some were associated with several); 174 of these have not previously been reported to be associated with lipid levels. Complete results are available in Tables II through V in the Data Supplement, and the level of overlap between CpGs associated with the 4 lipid fractions is depicted in Figure IXa in the Data Supplement.

In secondary analyses additionally adjusted for BMI, 80% (32/40), 87% (20/23), 13% (14/110), and 61% (17/28) of the CpG sites associated in the primary model with TC, LDL-C, HDL-C, and triglycerides, respectively, were significantly associated in the corresponding BMI-adjusted lipid model at a methylome-wide significant threshold (*P*<1.08E-7; Volcano plots in Figures I through IV in the Data Supplement). Associations of methylation with lipid levels after adjustment for BMI occurred at 80 unique CpGs (annotated to 60 unique genes). In these BMI-adjusted analyses, we found 9 CpG sites associated with lipid levels that were not significantly associated in the primary analyses (complete results available in Tables VI through IX in the Data Supplement; Figure IXb in the Data Supplement).

We then attempted to replicate the associations at the 193 CpG sites significantly associated with at least 1 lipid trait (in models without or with BMI adjustment) in 3 independent cohorts (≤2025 individuals) with DNA methylation from whole blood (LBC1936 and LBC1921) or CD4^+^ T cells (GOLDN). At a Bonferroni-corrected α threshold of 0.05 (taking the number of tests per lipid trait into account) and taking direction of effect into account, 5 (13%), 1 (4%), 11 (10%), and 19 (68%) of the CpG sites associated with TC, LDL-C, HDL-C and triglycerides, respectively, in the primary analysis replicated in a meta-analysis of these 3 independent cohorts (Table 1). When only considering the 10 most associated CpGs in the discovery for each lipid trait, the replication rate was considerable higher (30%, 10%, 40%, and 90% for TC, LDL-C, HDL-C, and triglycerides, respectively). Comparison of effect sizes between discovery and replication for all CpGs significant in the discovery stage revealed a high degree of overall concordance between the β coefficients (Pearson correlation coefficients 0.78, 0.67, 0.71, and 0.88, for TC, LDL-C, HDL-C, and triglycerides, respectively), indicating a high level of agreement even for CpGs that did not replicate at the *P* value threshold (Figure X in the Data Supplement). Comparison of effect sizes between discovery and each of the individual replication cohorts for all CpGs significant in the discovery is included in Figures XI through XIV in the Data Supplement. In secondary analyses adjusted for BMI in the external cohorts, we replicated 4 (13%), 1 (5%), 2 (14%), and 12 (71%) of the CpG sites associated with TC, LDL-C, HDL-C, and triglycerides, respectively (Table 1). In total, 33 CpGs replicated in the primary or secondary model (representing 55 associations as some CpGs were associated with several lipid traits). Twenty-five of these have not previously been reported to be associated with lipids in DNA methylation studies (Table 1; Table X in the Data Supplement). Ten of the lipid-associated CpGs (including 5 of the novel CpGs) have previously been associated with adiposity (BMI and waist circumference), glycemic traits (fasting insulin and insulin resistance by homeostasis model assessment), or type 2 diabetes mellitus in blood cell–derived DNA methylation data (Table X in the Data Supplement). We tested whether associations in blood could also be detected in another tissue using DNA methylation data from abdominal SAT from the MuTHER study^25^ (Tables II through IX in the Data Supplement). Less than half of HDL-C (40%) and triglyceride-associated (46%) sites were associated in SAT, and more than half of HDL-C sites were in opposite directions in blood and adipose tissue, indicating that there may be independent regulatory effects across tissue types.

**Table 1.**
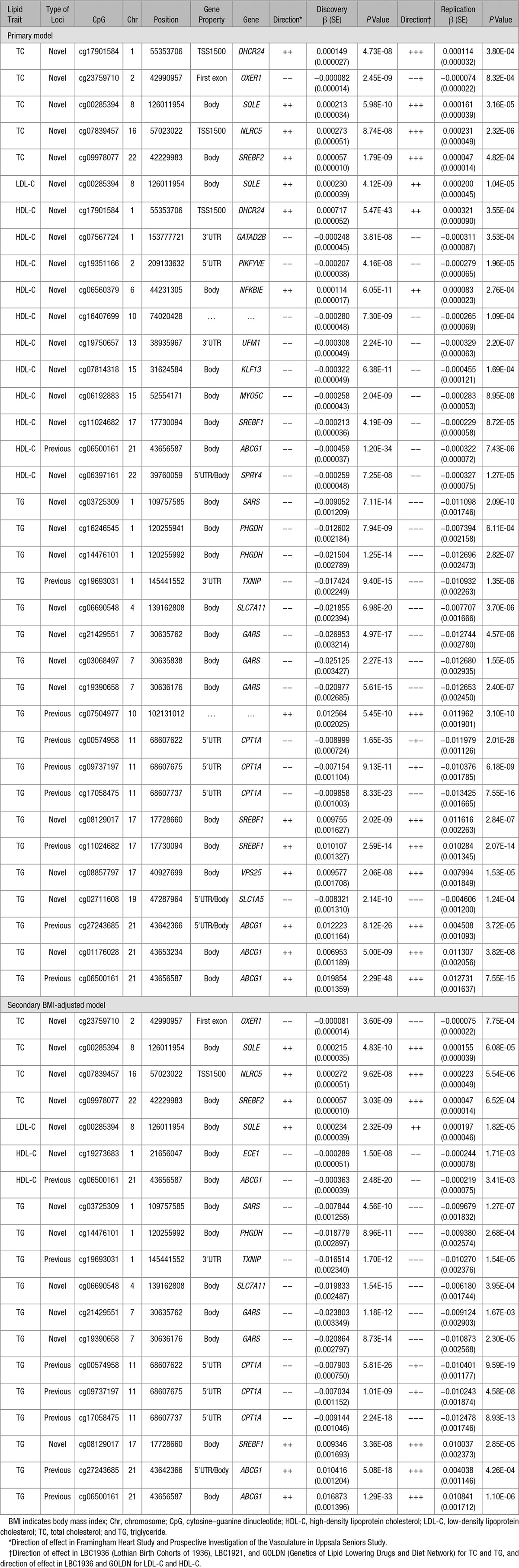
Lipid-Associated CpG Sites Replicated in Independent Cohorts With Whole Blood or CD4^+^ T Cells DNA Methylation

In addition, fixed effects meta-analyses across all 5 cohorts were performed for each lipid trait, identifying additional differentially methylated candidate regions that may play a role in lipid levels (Tables XI and XII in the Data Supplement), but that carry lesser weight given the lack of independent replication. Using the results of these meta-analyses, we investigated whether methylation at 15 CpGs associated with lipids in 2 recent publications^38,39^ also was associated with the same lipid traits in our study. We found 12 (80%) CpGs reported in previous studies to be associated with the same lipid traits in our study (Table 2), highlighting the high degree of between-study replicability of lipid–methylation associations. Interestingly, the intergenic CpG cg07504977 associated with triglycerides in both our study and the previous study lies in an active regulatory region (DNAse I hypersensitivity site and H3K27Ac mark) <10 kb distal to stearoyl-CoA desaturase (delta-9-desaturase). This gene plays an important role in the metabolism of dietary saturated fatty acids, a function that is critical for triglycerides synthesis and that has been shown to be disturbed in metabolic disease.^40^ However, in our study, methylation at cg07504977 was not associated with expression of stearoyl-CoA desaturase in whole blood.

**Table 2.**
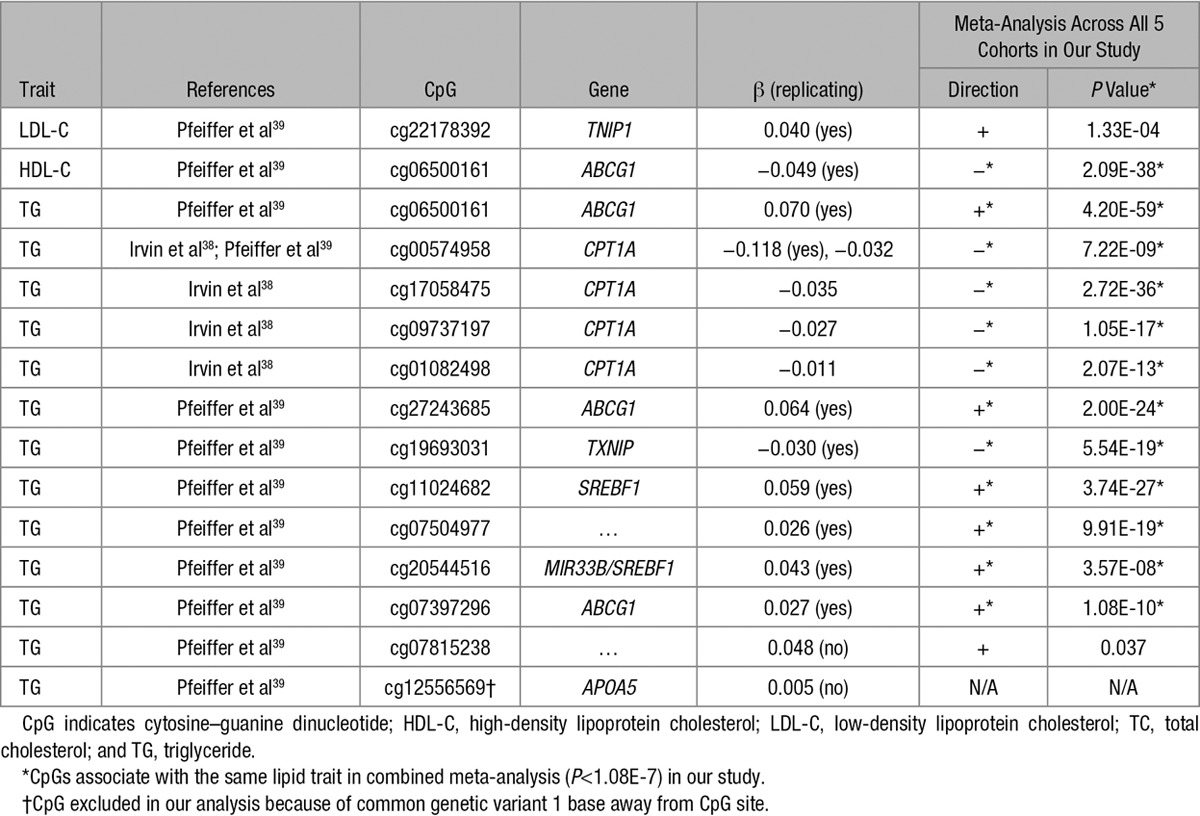
Associations of Lipid Levels With Methylation at CpGs Previously Reported to be Associated with Lipids

Many lipid-associated CpGs in our study were annotated to genes in loci highlighted in GWAS of cardiovascular traits, including lipids (*AMPD3*, *APOB*, *FADS2*, *GALNT2*, *LDLR*, *MYLIP*, and *TRIB1*), waist:hip ratio (*CBX3*, *KLF13*, and *LY86*), BMI (*ADCY3*), adiponectin (*TRIB1*), type 2 diabetes mellitus (*PTPRD*), and CHD (*APOB* and *LDLR*; Table XIII in the Data Supplement).

### Functional Annotation of Lipid-Associated CpGs

We explored the functional roles of the 193 CpGs associated with lipid traits by investigating their genomic locations with respect to genes, CpG islands, and functional regulatory elements. Lipid-associated CpGs were less commonly located in CpG islands (*P*=1.01E-15) and promoters (*P*=5.82E-04), when compared with all CpGs on the array (Figure XV in the Data Supplement). The observation that differential DNA methylation in relation to chronic human disease traits (as opposed to cancer) is less likely to be seen at promoters and CpG islands has been previously reported.^25^ To further explore the regulatory activity of identified loci, we examined the overlap of the 193 lipid-associated CpGs with functional regulatory elements across cell types using RegulomeDB.^41^ About 14% of sites showed strong evidence of being located in a functional regulatory region (RegulomeDB score 1a-2c; Tables II through IX in the Data Supplement); this was not more than expected by chance (*P*=0.83).

To further the *in silico* identification of relevant affected tissues, we used the eFORGE tool (http://eforge.cs.ucl.ac.uk/), which determines whether the identified CpGs are enriched in DNAse I hypersensitivity site hotspots in specific tissue types (Figure XVI in the Data Supplement). Our identified CpGs were in active DNAse I hypersensitivity site hotspots across a range of tissue types in ENCODE and Epigenome Roadmap Consortium tissue sets (FDR *Q* value <0.01), specifically blood, liver, muscle, heart, and epithelium (adipose tissue is not represented in this tool). Notably, the identified CpGs were not in DNAse I hypersensitivity site hotspots in nervous tissue (brain, cerebellum, hippocampus, and nervous), gastrointestinal tissue (colon, kidney, pancreas, and pancreatic duct), bone tissue, and eye tissue.

To place our findings in a broader biological context, we performed gene set enrichment analysis^31,32^ for genes annotated to the 193 CpGs associated with lipid levels. For TC, the pathway analyses revealed enrichment in processes relating to sterol, lipid, and cholesterol metabolism and biosynthesis (FDR=0.0029–0.037), indicating that DNA methylation sites associated with cholesterol primarily affect processes directly relating to lipid production and metabolism (Tables XIV through XVI in the Data Supplement). For triglycerides, the pattern was different because metabolism of amino acids was highlighted in the pathway analyses (FDR=0.034). No significant enrichment in pathways was observed in analysis of genes annotated to CpG sites associated with LDL-C or HDL-C. When restricting the enrichment analyses to genes annotated to replicating CpGs or to those where methylation levels were associated with gene expression of their respective genes, we observed similar results (Tables XIV through XVI in the Data Supplement), with the exception of HDL-C, which now showed significant enrichment in lipid metabolism (FDR=0.0056–0.04).

### Genetic Regulation of Lipid-Associated DNA Methylation

To assess the role of genetic variation in controlling lipid-related DNA methylation changes, we studied the association of sequence variants in *cis* with methylation levels at lipid-associated CpGs (*cis*-meQTLs). Mapping of *cis*-meQTLs (SNPs in a 100 kb window around CpG sites) was performed in the FHS cohort (n=2246) with subsequent replication of lead meQTLs in the PIVUS cohort (n=775). In agreement with previous studies,^6,25^ we found a large proportion of CpG sites to associate with common SNPs in *cis*. We found 123 out of 193 (64%) lipid-associated CpG sites to be at least partly regulated by genetic sequence variation in *cis* (*P*<1E-04); 60 of these replicated in PIVUS (at *P*<4.071E-04; Table XVII in the Data Supplement).

We investigated whether the 123 significant lead meQTL SNPs or their proxies (*r*^*2*^>0.8) were over-represented among SNPs with nominally significant associations (*P*<0.05) in GWAS meta-analyses from the CARDIoGRAM consortium for CHD^42^ and the Global Lipids Genetics consortium for lipid levels.^4^ We found evidence of enrichment (applying a 1-sided Fisher exact test) of nominally significant associations for CHD (*P*=7.04E-4), TC (*P*=4.36E-3), HDL-C (*P*=8.3E-3), and triglycerides (*P*=2.9E-5) among the *cis*-meQTL lead SNPs (or proxies). Furthermore, we found the lead *cis*-meQTL SNP (rs563290) of cg05337441 (associated with LDL-C in discovery, *P*=4.5E-8 but not surviving Bonferroni cutoff threshold in replication, *P*=1.7E-2), located in an intron of *APOB*, to be associated with LDL-C in GWAS^43^ and to be a highly correlated proxy (*r*^2^=1) of genome-wide significant GWAS index SNPs (rs515135 and rs562338; located ≈20 kb upstream of the *APOB* transcription start site) in LDL-C meta-analyses.^44,45^ This *cis*-meQTL proxy for *APOB* locus methylation (rs515135) is also associated with CHD at a genome-wide level of significance (*P*=1.8E-10) from the CardiogramC4D consortium data.^42^

### The Impact of Lipid-Associated CpGs on Gene Expression

Examining gene expression in relation to DNA methylation in blood from participants in the FHS, we investigated whether methylation levels at lipid-associated CpGs were associated with mRNA expression levels of nearby genes (±500 kb). We found 29 CpGs (out of 193 tested; 15%) to be associated with expression in blood of at least 1 adjacent gene (FDR<0.05; 36 CpG–expression pairs in total; Table XVIII in the Data Supplement). For the majority (86%) of these associations, levels of methylation and expression were inversely correlated. For 17 of these 29 CpGs (59%), there was also a significant *cis*-meQTL. The lead meQTL SNP was significantly associated with both methylation and gene expression (FDR<0.05) for 12 of 36 CpG–expression pairs (29 unique CpGs), suggesting that the genotype may affect both methylation and expression. This was the case for the following genes: *CHSY1* (cg24002003), *DHCR24* (cg17901584), *ECE1* (cg19273683), *IL18R1* (cg05295703), *IL1RL1* (cg05295703), *KANK2* (cg01751802), *LDLR* (cg26313301), *PHGDH* (cg14476101, cg16246545), *PRKD2* (cg22304262), *SREBF1* (cg08129017), and *SREBF2* (cg09978077). For the remaining 6 CpG–expression pairs, the meQTL SNP was associated with methylation (FDR<0.05) but not with expression (FDR >0.05) as presented in Table XVIII in the Data Supplement.

### Detailed Characterization of Lipid CpG Sites Using Metabolomics

To further characterize functional relevance of lipid-associated CpG sites, we tested levels of methylation at the 193 CpGs for association with 229 serum metabolites in the PIVUS cohort.^46^ We found 29 of the lipid-associated CpGs to be associated with at least 1 metabolite (FDR<0.01; Table XIX in the Data Supplement). As expected, the majority of the associations were between a lipid-related CpG site and various lipid-derived metabolites (Figure XVII in the Data Supplement). Most associations were observed with cg17901584 in the promoter of *DHCR24* (associated with TC, HDL-C, and triglycerides) and with sites in the promoter of *ABCG1* (associated with HDL-C and triglycerides), highlighting the central role for these genes in lipid metabolism. Metabolites associated with methylation of the *DHCR24* promoter included a derivate of cinnamic acid, recently shown to be associated with a lower risk of incident CHD events.^46^ Methylation at the *ABCG1* locus was associated with specific ceramides and sphingomyelins, which have been implicated in the development of atherosclerosis and CHD.^47,48^

### Association of Lipid-Associated CpGs With Disease Outcomes

**Figure 2.**
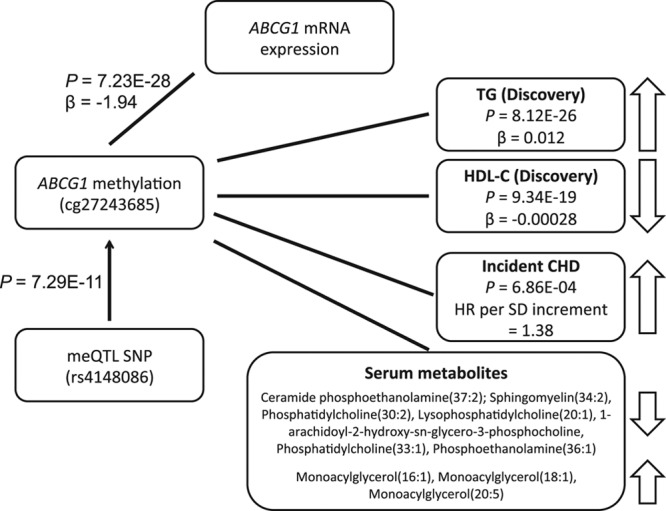
Associations at the *ABCG1* locus. CHD indicates coronary heart disease; HDL-C, high-density lipoprotein cholesterol; meQTL, methylation quantitative trait locus; SNP, single-nucleotide polymorphism; and TG, triglyceride.

We investigated whether the 33 replicating lipid-associated CpG sites were also associated with incident CHD events during an 8-year follow-up in the FHS (number of CHD events=115) and a 10-year follow-up in PIVUS (number of CHD events =78) using multivariable Cox proportional hazard models. Methylation levels at *ABCG1* (cg27243685) were significantly associated (Bonferroni-corrected α<0.05, nominal *P*<1.52E-03) with CHD in a meta-analysis of FHS and PIVUS (hazard ratio per SD increment=1.38; 95% confidence interval, 1.15–1.66; *P*=6.86E-04; Table XX in the Data Supplement). We found the relationship of methylation at cg27243685 with triglycerides and risk of CHD to be directionally consistent with the expected based on previous studies of lipid levels and CHD risk.^1–3^ Hypermethylation at cg27243685 in the 5′-UTR of *ABCG1*—that was associated with decreased expression of *ABCG1* (Table XVIII in the Data Supplement)—was associated with higher triglycerides and lower HDL-C, as well as increased risk for CHD (Figure 2). This *ABCG1* locus (cg27243685) was also highlighted in the previous sections as containing a *cis*-meQTL and being associated with metabolites. This illustrates an example of a pathway linking genetic variant to perturbed DNA methylation, altered expression levels, circulating metabolites, lipid levels (triglycerides and HDL-C), and risk of CHD (Figure 2).

## Discussion

In this study, we aimed to identify epigenetic variation associated with serum lipid concentrations, which are among the most established risk factors for CVD. We report findings of a genome-wide scan of blood DNA methylation in relation to circulating lipid levels from ≤2306 individuals with independent external replication in ≤2025 additional individuals. We extend the findings of published literature on the association of differential DNA methylation with circulating lipids^38,39,49,50^ by examining larger discovery and replication samples and by examining the association of methylation at the associated CpGs with gene expression, intermediate metabolites, and incident CHD. We have made several novel observations about the role of DNA methylation in the regulation of lipids and risk of CVD and highlight 3 important contributions. First, we identified novel replicated loci of differential methylation in blood associated with circulating lipid levels that may represent potential therapeutic targets. Second, we describe the overlap of methylation and GWAS SNPs and identify a potential mechanism of a known LDL-C–related GWAS variant at the *APOB* locus acting as a *cis*-meQTL on LDL-C–related differential methylation at cg05337441, intronic to *APOB*. Third, we identify HDL-C–related and triglyceride-related differential methylation at the *ABCG1* locus (cg27243685) to be associated with expression of a gene involved in reverse cholesterol transport (*ABCG1*), metabolites that influence reverse cholesterol transport (sphingomyelins), and subsequently to be associated with a 38% higher risk of incident CHD per SD increase in methylation.

We found methylation at 193 CpG sites to be associated with lipid levels and replicated 33 of these in 3 independent cohorts with data on DNA methylation in blood and T cells. Many of the differentially methylated loci associated with LDL-C, triglycerides, and to a lesser degree HDL-C, were independent of adjustment for BMI. Twenty-five of the 33 replicated CpGs have not been previously reported to be associated with lipid levels.^38,39^ Novel sites included those near genes with a known function in cholesterol metabolism (*DHCR24, SREBF2*, and *SQLE*) and with a possible role in atherosclerosis (endothelin-converting enzyme-1).^51,52^ The novel genes identified warrant further research as potential targets for perturbation to reduce dyslipidemia.

When exploring whether methylation at lipid-associated CpGs has also been associated with related cardiometabolic traits, we found overlap with associations for adiposity (near genes *ABCG1*, *CPT1A*, *DHCR24*, *KLF13*, *MYO5C*, *PHGDH*, *SREBF1*, and *VPS25*),^53–55^ glycemic traits (near *ABCG1,*)^56^ and type 2 diabetes mellitus (near genes *SREBF1*, *ABCG1,* and *TXNIP*).^57^ In addition, we observed associations of circulating lipids with DNA methylation levels at CpGs near genes previously reported to be associated with lipids, other cardiovascular traits, and CVD events in GWAS.

Further, pathway analyses, including genes annotated to lipid-associated CpGs, showed enrichment in pathways involved in lipid, sterol, and cholesterol metabolic and biosynthesis processes for cholesterol-related CpGs, whereas amino acid metabolism pathways were enriched for triglyceride-associated CpGs. These observations highlight the different biological mechanisms underlying changes in genomic regulation observed in association with TC and TGs.

We identified genetic drivers of lipid-associated CpGs in blood through integration with SNPs in *cis*-meQTLs analyses. At 64% of the lipid-associated CpGs, the effect is determined in part by genotype. GWAS SNPs for lipids and CHD were enriched among the *cis*-meQTL SNPs of lipid-associated CpGs. Further, we observed association with expression levels of adjacent genes for 15% of the CpGs, which indicates possible mechanisms of effect through changes in transcription. For 17 of the lipid-related CpGs where there was an association with expression levels of an adjacent gene, there was also a significant *cis*-meQTL. For the majority of these, the genotype affected both methylation and gene expression. In these instances, our data provide evidence linking multiple steps from genetic variants affecting DNA methylation, to modulation of gene expression to effects on circulating lipid levels. For example, at the *ABCG1* locus, we observed that the minor allele at intronic variant rs4148086 was associated with increased methylation at cg27243685. This methylation marker, which is located at the south shelf of a CpG island in the 5′-UTR region of *ABCG1*, was associated with decreased expression of *ABCG1* in blood, increased triglyceride levels (even after adjustment of BMI and regulated both by blood and SAT methylation), and increased risk of new-onset CHD. Methylation in this locus (at cg06500161) has previously been associated with prevalent myocardial infarction.^39^ The *ABCG1* gene product functions in the efflux of cholesterol from lipid-loaded macrophages to HDL-C.^58^ However, the functional basis for association to levels of triglycerides in blood circulation is unclear. Although circulating HDL-C levels has been largely disproven as a causal factor for CHD,^2^ the importance of cholesterol efflux function in CHD risk is an emerging topic of discussion.^59^ In addition to cholesterol, ABCG1 mediates the efflux of sphingomyelin and phosphatidylcholine, and the cholesterol efflux by ABCG1 has been demonstrated to have some dependence on sphingomyelin concentrations.^60,61^ Sphingomyelins have been implicated in the development of atherosclerosis and CHD.^48,62^ In our study, methylation in the *ABCG1* locus was also associated with specific sphingomyelins and ceramides (also implicated in CHD^47^). Methylation at CpG sites in the *ABCG1*, as well as the *DHCR24* loci, was also associated with a large number of other lipid-related metabolites in blood, further highlighting the central role for these genes in processes relating to lipid metabolism and development of CVD.

The main strengths of this study include the large sample size of the genome-wide DNA methylation and ≤10 years of follow-up allowing analyses of incident CHD end points. In addition, inclusion of several other types of functional genomics data (gene expression and metabolites) helped us to draw more precise conclusions on the links between methylation and circulating lipid levels. We replicated a large fraction of previously reported associations of methylation and lipid levels, providing assurance that associations of methylation with lipid levels are reliable across different studies and indicate that also the novel findings reported may indeed represent true findings.

The study also has limitations. Blood-derived cells, although easily accessible and good for biomarker discovery, may not be the most relevant tissue for drawing biological conclusions. Our validation in adipose tissue reveals that at least a proportion of the observed associations are shared across tissues. The cross-sectional design does not allow us to determine the causal relationship between lipid and DNA methylation. Our analysis of lipid-associated CpGs with incident disease indicates the relevance of methylation in at least one of these CpGs for disease pathophysiology. Further, a relatively low proportion of our findings could be robustly validated in the replication stage. However, it should be noted that we observed a high level of agreement of β coefficients even for CpGs that did not formally replicate at the *P* value threshold. This indicates that the low replication rate may be because of smaller sample size in the replication stage, particularly for LDL-C and HDL-C, giving reduced power, especially in the light of our strict criteria for replication (which was chosen to minimize false-positive findings). In addition, if the differentially methylated CpGs identified in discovery from whole blood did not also occur in CD4^+^ T cells, we would not expect to see replication in the GOLDN replication cohort that assayed DNA from cell-sorted CD4^+^ T cells. Furthermore, cholesterol panels from the LBC cohort were obtained in a nonfasting state and may have reduced our ability to replicate findings. Finally, transcriptomic and metabolomic data were not available in every cohort, and, therefore, we were not able to demonstrate similar findings in each participating study.

In conclusion, we report novel associations of DNA methylation with lipid levels. We identify links between genetic variation underlying lipids and CHD to differential DNA methylation. We also highlight HDL-C–related and triglyceride-related differential methylation and expression of a reverse cholesterol transporter, *ABCG1*, and the association with an increased risk of incident CHD. Our findings highlight established and novel targets and mechanisms that can be used as a starting point for potential new treatments for dyslipidemia and CVD.

## Acknowledgments

The FHS (Framingham Heart Study), PIVUS (Prospective Investigation of the Vasculature in Uppsala Seniors Study), LBC (Lothian Birth Cohorts), and GOLDN (Genetics of Lipid Lowering Drugs and Diet Network) studies thank the cohort participants and team members who contributed to these studies. Data on coronary artery disease/myocardial infarction have been contributed by CARDIoGRAMplusC4D investigators and have been downloaded from http://www.cardiogramplusc4d.org/. The views expressed in this article are those of the authors and do not necessarily represent the views of the National Heart, Lung, and Blood Institute (NHLBI); the National Institutes of Health (NIH) or the US Department of Health and Human Services.

## Sources of Funding

FHS (Framingham Heart Study) is funded by National Institutes of Health (NIH) contract N01-HC-25195 and HHSN268201500001I and administered by Boston University. The laboratory work for this investigation was funded by the Division of Intramural Research, National Heart, Lung, and Blood Institute (NHLBI), NIH, and an NIH Director’s Challenge Award (Dr Levy, Principal Investigator). The analytic component of this project was funded by the Division of Intramural Research, NHLBI, and the Center for Information Technology, NIH, Bethesda, MD. This study used the computational resources of the Biowulf system at the NIH, Bethesda, MD (https://hpc.nih.gov/). Dr Mendelson is partly supported by the Tommy Kaplan Fund, Boston Children’s Hospital. Dr Liang is partially supported by NIH grant P30 DK46200. Dr Ingelsson is supported by Knut and Alice Wallenberg (KAW) Foundation, Swedish Research Council (VR; grant no. 2012-1397), Swedish Heart-Lung Foundation (20120197) NIH grants 1R01DK106236-01A1 and 1R01HL135313-01. Genome-wide DNA methylation profiling in PIVUS was funded by the Uppsala University Hospital (ALF-medel) and was performed by the SNP&SEQ Technology Platform in Uppsala. The facility is part of the National Genomics Infrastructure Sweden and Science for Life Laboratory. The SNP&SEQ Platform is also supported by the VR and the KAW Foundation. Phenotype collection in the LBC1921 (Lothian Birth Cohorts of 1921) study was supported by the UK Biotechnology and Biological Sciences Research Council (BBSRC), The Royal Society and The Chief Scientist Office of the Scottish Government. Phenotype collection in the LBC1936 (Lothian Birth Cohorts of 1936) study was supported by Age UK (The Disconnected Mind project). Methylation typing was supported by the Centre for Cognitive Ageing and Cognitive Epidemiology (CCACE; Pilot Fund award), Age UK, The Wellcome Trust Institutional Strategic Support Fund, The University of Edinburgh, and The University of Queensland. Drs Marioni, Starr, and Deary are members of the University of Edinburgh CCACE. CCACE is supported by funding from the BBSRC, the Medical Research Council and the University of Edinburgh as part of the cross-council Lifelong Health and Wellbeing initiative (MR/K026992/1). Research reported in this publication was supported by National Health and Medical Research Council (NHMRC) project grant 1010374 and an NHMRC Fellowship to Dr McRae (1083656). The GOLDN (Genetics of Lipid Lowering Drugs and Diet Network) study was supported by NIH National Heart, Lung and Blood Institute grant R01 HL104135-01. The MuTHER study was funded by the Wellcome Trust; European Community’s Seventh Framework Programme (FP7/2007–2013). The study as part of TwinsUK also receives support from the Medical Research Council, European Union, National Institute for Health Research-funded BioResource, Clinical Research Facility and Biomedical Research Centre based at Guy’s and St Thomas’ NHS Foundation Trust in partnership with King’s College London. Dr Spector is a holder of an European Research Council Advanced Principal Investigator award.

## Disclosures

Erik Ingelsson is an advisor and consultant for Precision Wellness, Inc., and advisor for Cellink for work unrelated to the present project.

## Supplementary Material

## CLINICAL PERSPECTIVE

Serum lipid levels are among the most established risk factors for cardiovascular disease, the leading cause of death globally. In this study, we report on the relations of circulating serum lipids with epigenetic marks and also provide evidence of a role for epigenetics in cardiovascular disease development. We present findings from a genome-wide scan of blood DNA methylation in relation to circulating lipid levels from 2306 individuals with independent external replication in 2025 individuals. We have made several novel observations about the role of DNA methylation in the regulation of lipids and risk of cardiovascular disease and highlight 3 important contributions: (1) we identify novel replicated loci of differential methylation in blood associated with circulating lipid levels that may represent potential therapeutic targets, (2) we describe the overlap of methylation and genome-wide association studies single-nucleotide polymorphisms and identify a potential mechanism of a known low-density lipoprotein cholesterol–related and coronary heart disease–related single-nucleotide polymorphism from genome-wide association studies at the *APOB* locus acting as a *cis*-methylation quantitative trait locus on low-density lipoprotein cholesterol–related differential methylation at a site intronic to *APOB*, and (3) we identify triglyceride- and high-density lipoprotein cholesterol–related differential methylation at the *ABCG1* locus to be associated with expression of a gene involved in reverse cholesterol transport (*ABCG1*), metabolites that influence reverse cholesterol transport (sphingomyelins), and subsequently to be associated with a 38% higher risk in incident coronary heart disease events. We think that our findings provide important insights into the contributions of epigenetics in circulating lipids and cardiovascular disease and may provide insights to novel therapeutic targets.
