# Supplementary material for: Epigenetic Patterns in Blood Associated With Lipid Traits Predict Incident Coronary Heart Disease Events and Are Enriched for Results From Genome-Wide Association Studies
Source: Circ Cardiovasc Genet. 2017 Feb 20;10(1):e001487. doi: 10.1161/CIRCGENETICS.116.001487 (PMC5331877; doi:10.1161/CIRCGENETICS.116.001487)
Supplement: Supplementary file 1 [file hcg-10-e001487-s001.pdf]

SUPPLEMENTAL MATERIAL

Supplementary Figures

Supplementary Fig. 1. Volcano plots of TC model 1 and 2 in the discovery *Page 4*
Supplementary Fig. 2. Volcano plots of LDL-C model 1 and 2 in the discovery *Page 5*
Supplementary Fig. 3. Volcano plots of HDL-C model 1 and 2 in the discovery *Page 6*
Supplementary Fig. 4. Volcano plots of TG model 1 and 2 in the discovery *Page 7*
Supplementary Fig. 5. Manhattan plot of genome-wide analysis of TC (model 1) in the discovery *Page 8*
Supplementary Fig. 6. Manhattan plot of genome-wide analysis of LDL-C (model 1) in the discovery *Page 9*
Supplementary Fig. 7. Manhattan plot of genome-wide analysis of HDL-C (model 1) in the discovery *Page 10*
Supplementary Fig. 8. Manhattan plot of genome-wide analysis of TG (model 1) in the discovery *Page 11*
Supplementary Fig. 9. Overlap of DNA methylation sites associated with the blood lipid levels in the discovery. *Page 12*
Supplementary Fig. 10. Comparison of effect size estimates between the discovery and replication *Page 13*
Supplementary Fig. 11. Comparison of effect size estimates for TC model 1 between the discovery and each of the individual replication cohorts *Page 14*
Supplementary Fig. 12. Comparison of effect size estimates for LDL-C model 1 between the discovery and each of the individual replication cohorts *Page 15*
Supplementary Fig. 13. Comparison of effect size estimates for HDL-C model 1 between the discovery and each of the individual replication cohorts *Page 16*
Supplementary Fig. 14. Comparison of effect size estimates for TG model 1 between the discovery and each of the individual replication cohorts *Page 17*
Supplementary Fig. 15. Location of lipid-associated CpGs in relation to CpG islands and genes. *Page 18*
Supplementary Fig. 16. Enrichment of lipid-associated CpGs in DHS hotspots from specific tissues using eFORGE tool *Page 19*
Supplementary Fig. 17. Overview of significant associations between lipid-associated CpGs and serum metabolites. *Page 20*

Supplementary Tables

Supplementary Table 1. Cohort characteristics *Page 21*
Supplementary Table 2. CpG sites associated with TC in discovery (P<1.08E-07). Sites replicating at Bonferroni-corrected alpha threshold 0.05 are highlighted in bold. *see separate xls*
Supplementary Table 3. CpG sites associated with LDL-C in discovery (P<1.08E-07). Sites replicating at Bonferroni-corrected alpha threshold 0.05 are highlighted in bold. *see separate xls*
Supplementary Table 4. CpG sites associated with HDL-C in discovery (P<1.08E-07). Sites replicating at Bonferroni-corrected alpha threshold 0.05 are highlighted in bold. *see separate xls*
Supplementary Table 5. CpG sites associated with TG in discovery (P<1.08E-07). Sites replicating at Bonferroni-corrected alpha threshold 0.05 are highlighted in bold. *see separate xls*
Supplementary Table 6. CpG sites associated with BMI-adjusted TC in discovery (P<1.08E-07). Sites replicating at Bonferroni-corrected alpha threshold 0.05 are highlighted in bold. *see separate xls*
Supplementary Table 7. CpG sites associated with BMI-adjusted LDL-C *see separate*

in discovery (P<1.08E-07). Sites replicating at Bonferroni-corrected alpha threshold 0.05 are highlighted in bold. *xls*

Supplementary Table 8. CpG sites associated with BMI-adjusted HDL-C in discovery (P<1.08E-07). Sites replicating at Bonferroni-corrected alpha threshold 0.05 are highlighted in bold. *see separate see separate xls*

Supplementary Table 9. CpG sites associated with BMI-adjusted TG in discovery (P<1.08E-07). Sites replicating at Bonferroni-corrected alpha threshold 0.05 are highlighted in bold. *see separate xls*

Supplementary Table 10. Association of replicated lipid-associated CpG sites with the same or other traits in published data *see separate xls*

Supplementary Table 11. CpG sites associated with TC, LDL-C, HDL-C or TG in combined meta-analysis at Bonferroni-corrected alpha threshold 0.05 (P<1.08E-07). *see separate xls*

Supplementary Table 12. CpG sites associated with TC, LDL-C, HDL-C or TG in combined meta-analysis at Bonferroni-corrected alpha threshold 0.05 (P<1.08E-07). Results in table from the secondary BMI-adjusted model. *see separate xls*

Supplementary Table 13. Genes annotated to lipid CpGs in GWAS loci of cardiovascular traits *Page 22*

Supplementary Table 14. Enriched biological categories among genes annotated to TC-associated CpGs (adjusted P-value <0.1) *Page 23*

Supplementary Table 15. Enriched biological categories among genes annotated to HDL-C associated CpGs (adjusted P-value <0.1) *Page 24*

Supplementary Table 16. Enriched biological categories among genes annotated to TG-associated CpGs (adjusted P-value <0.1) *Page 25*

Supplementary Table 17. Lipid-associated CpGs regulated by genetic sequence variants in *cis* (*P*<1E-04) in FHS and replication in PIVUS. *see separate xls*

Supplementary Table 18. Lipid-associated CpGs significantly associated with levels of expression of neighbouring genes in blood in FHS (FDR <0.05). *Page 26*

Supplementary Table 19. Association of lipid-associated CpGs sites significant in discovery with blood metabolites (FDR <0.01). *see separate xls*

Supplementary Table 20. Association of lipid-associated CpGs with incident CHD up to ten years after baseline in FHS and PIVUS. *Page 27*

Supplementary Methods

Cohort descriptions and sample collections *Page 28*

Genome-wide DNA methylation profiling *Page 30*

Association of DNA methylation levels in blood cell derived data with lipids *Page 31*

Genotyping and Imputation *Page 33*

Gene expression profiling *Page 33*

Metabolomic profiling *Page 34*

Supplementary References *Page 34*

A.

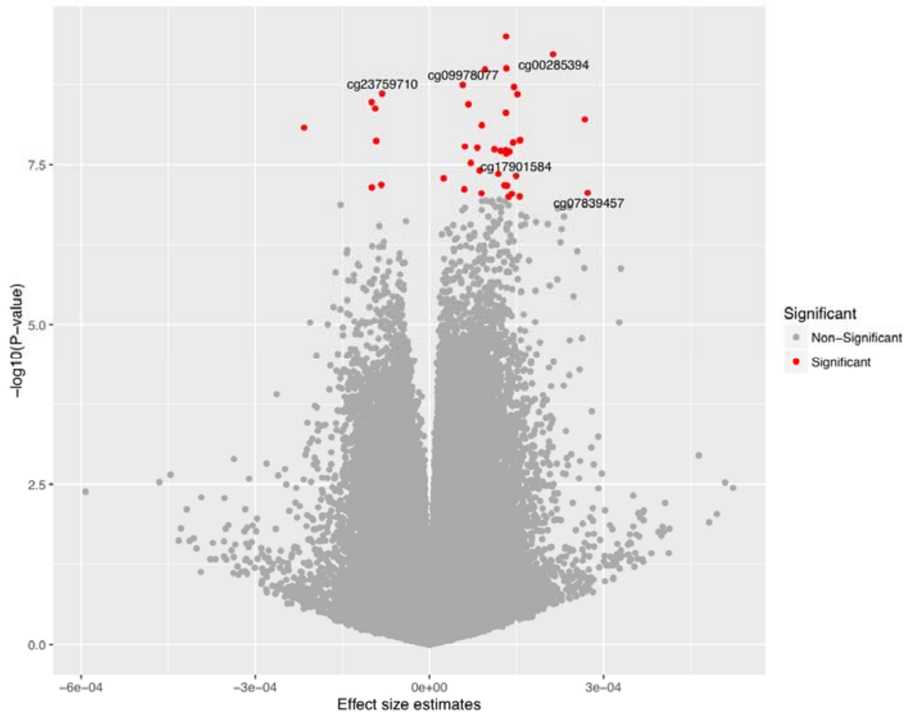

B.

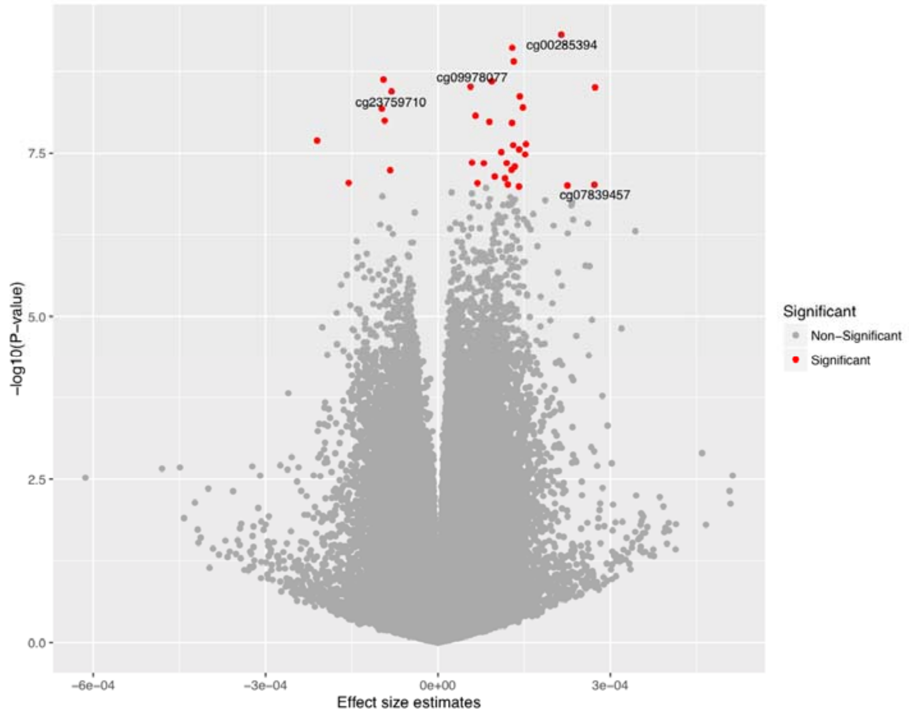

Supplementary Fig. 1. Volcano plots of TC models 1 and 2 in the discovery.
A. Results of meta-analysis of FHS and PIVUS (discovery) TC model 1. **B.** Results of meta-analysis of FHS and PIVUS (discovery) BMI-adjusted TC model 2. Significant associations are coloured in red. Replicating associations are labelled by CpG marker name in the plot.

A.

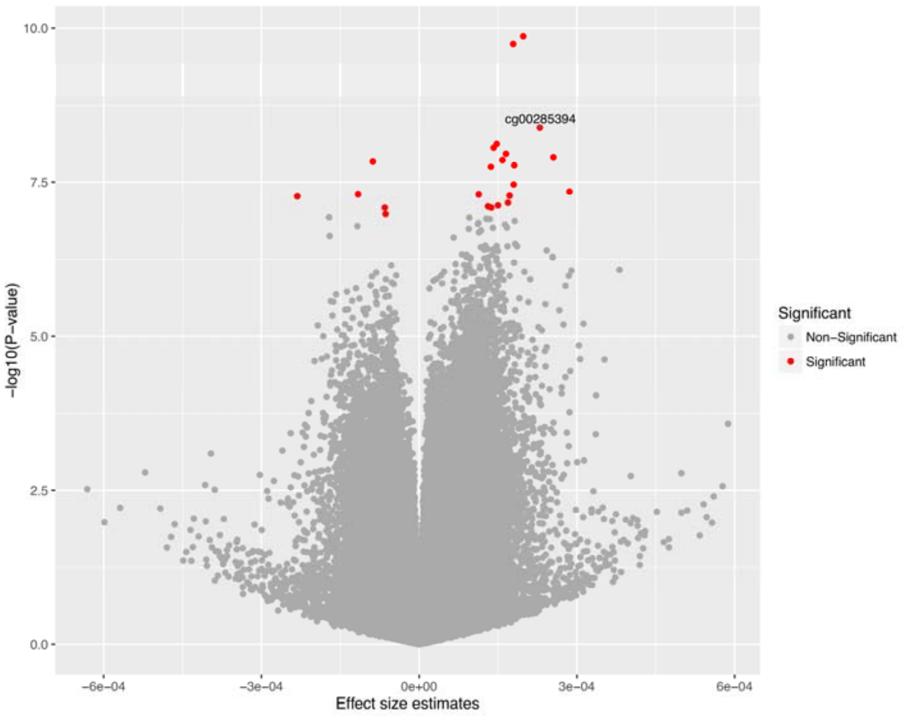

B.

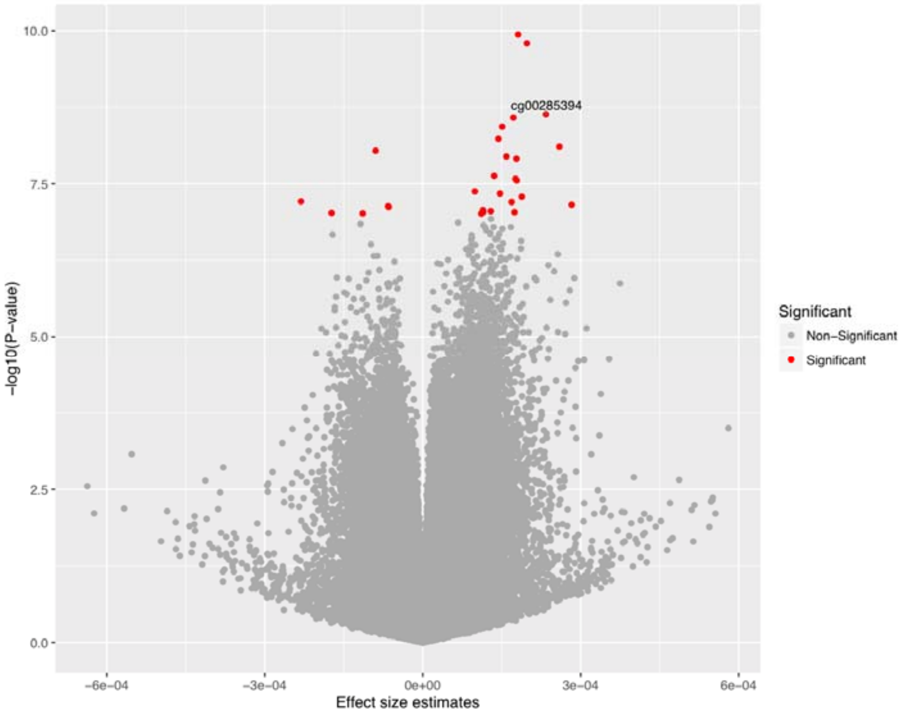

Supplementary Fig. 2. Volcano plots of LDL-C models 1 and 2 in the discovery.
A. Results of meta-analysis of FHS and PIVUS (discovery) LDL-C model 1. **B.** Results of meta-analysis of FHS and PIVUS (discovery) BMI-adjusted LDL-C model 2. Significant associations are coloured in red. Replicating associations are labelled by CpG marker name in the plot.

A.

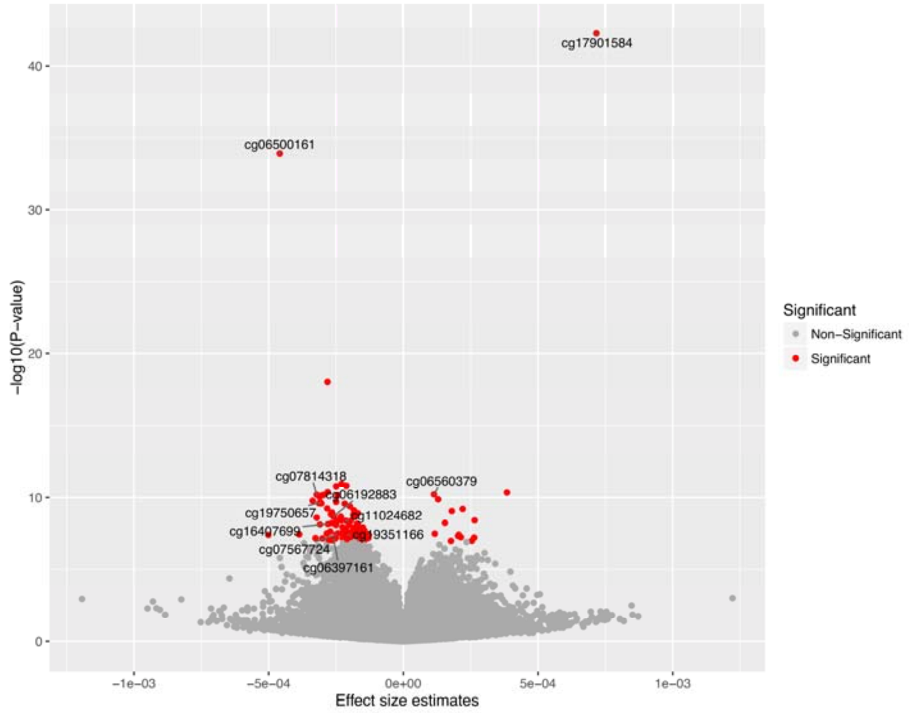

B.

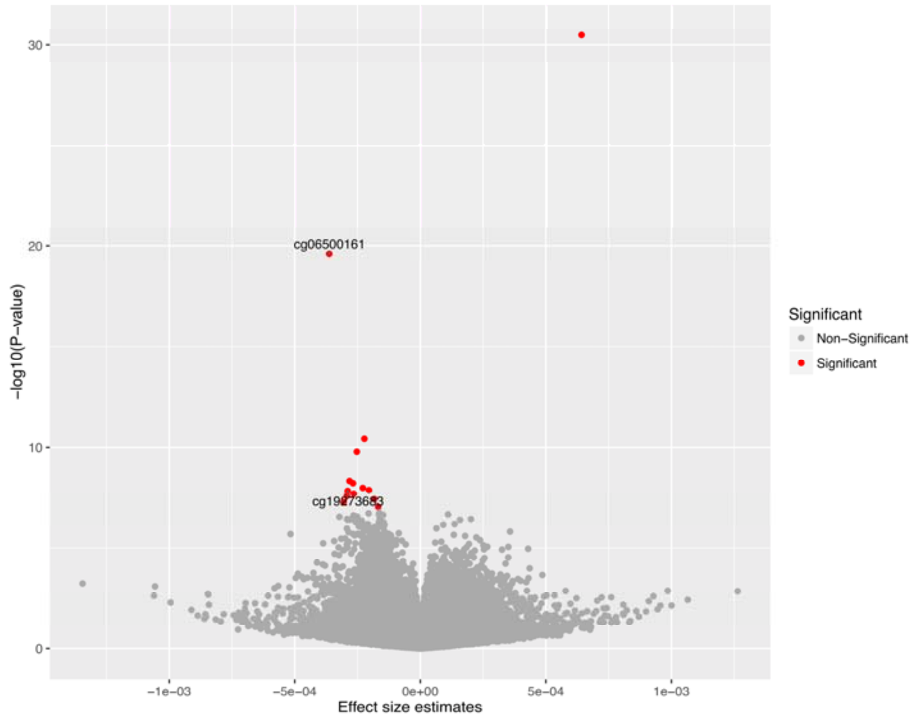

Supplementary Fig. 3. Volcano plots of HDL-C models 1 and 2 in the discovery.
A. Results of meta-analysis of FHS and PIVUS (discovery) HDL-C model 1. **B.** Results of meta-analysis of FHS and PIVUS (discovery) BMI-adjusted HDL-C model 2. Significant associations are coloured in red. Replicating associations are labelled by CpG marker name in the plot.

A.

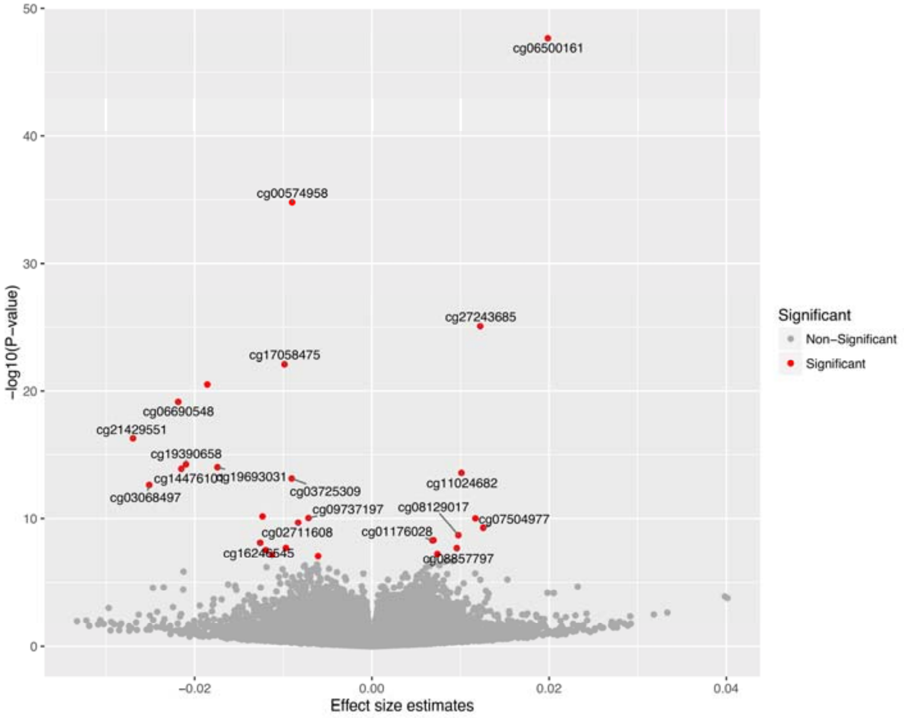

B.

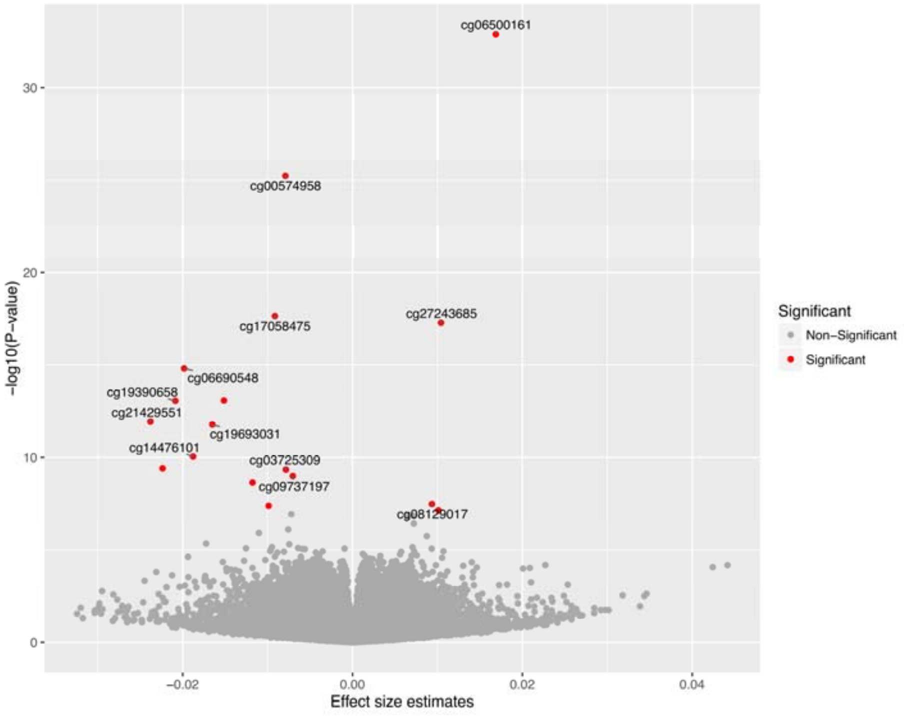

Supplementary Fig. 4. Volcano plots of TG models 1 and 2 in the discovery.
A. Results of meta-analysis of FHS and PIVUS (discovery) TG model 1. **B**. Results of meta-analysis of FHS and PIVUS (discovery) BMI-adjusted TG model 2. Significant associations are coloured in red. Replicating associations are labelled by CpG marker name in the plot.

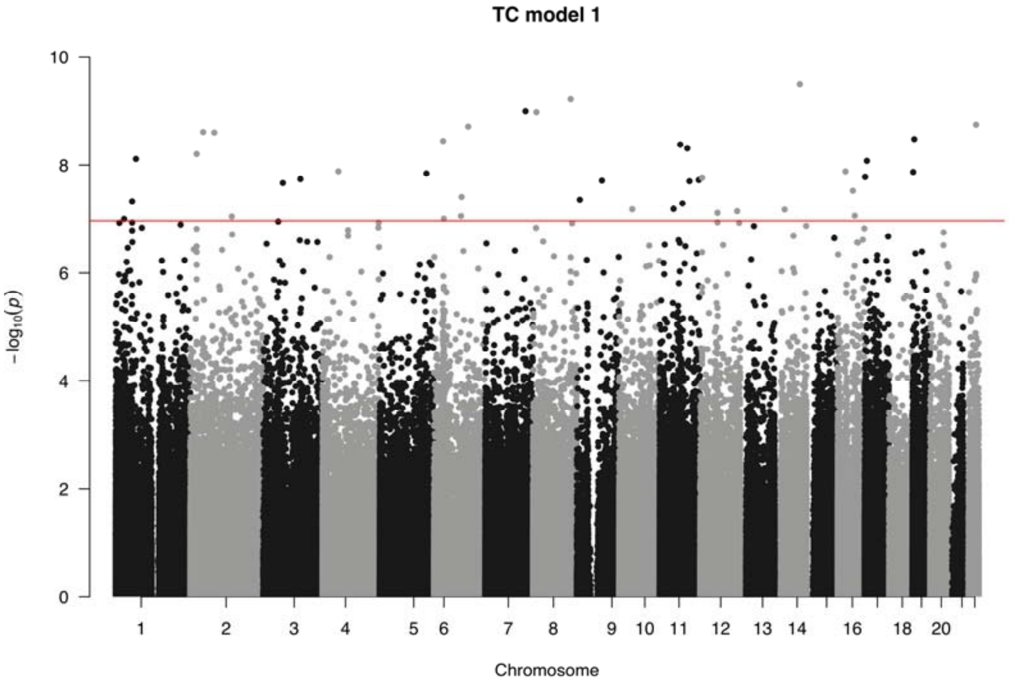

Supplementary Fig. 5. Manhattan plot of genome-wide analysis of TC (model 1) in the discovery. The red line indicates the methylome-wide significance level (*P*<108E-07).

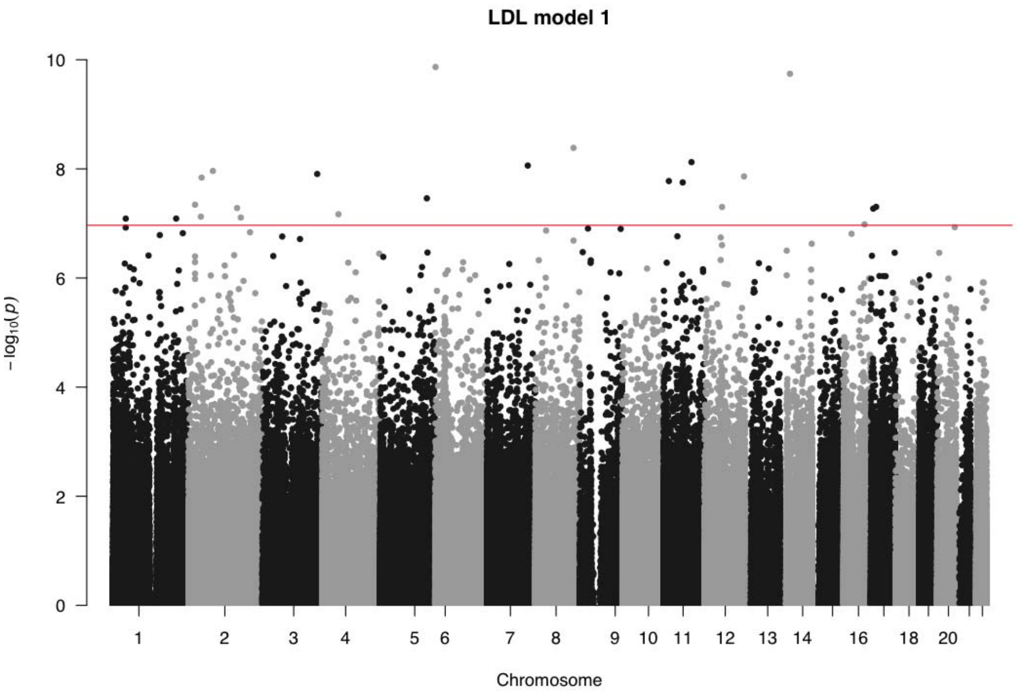

Supplementary Fig. 6. Manhattan plot of genome-wide analysis of LDL-C (model 1) in the discovery. The red line indicates the methylome-wide significance level (*P*<108E-07).

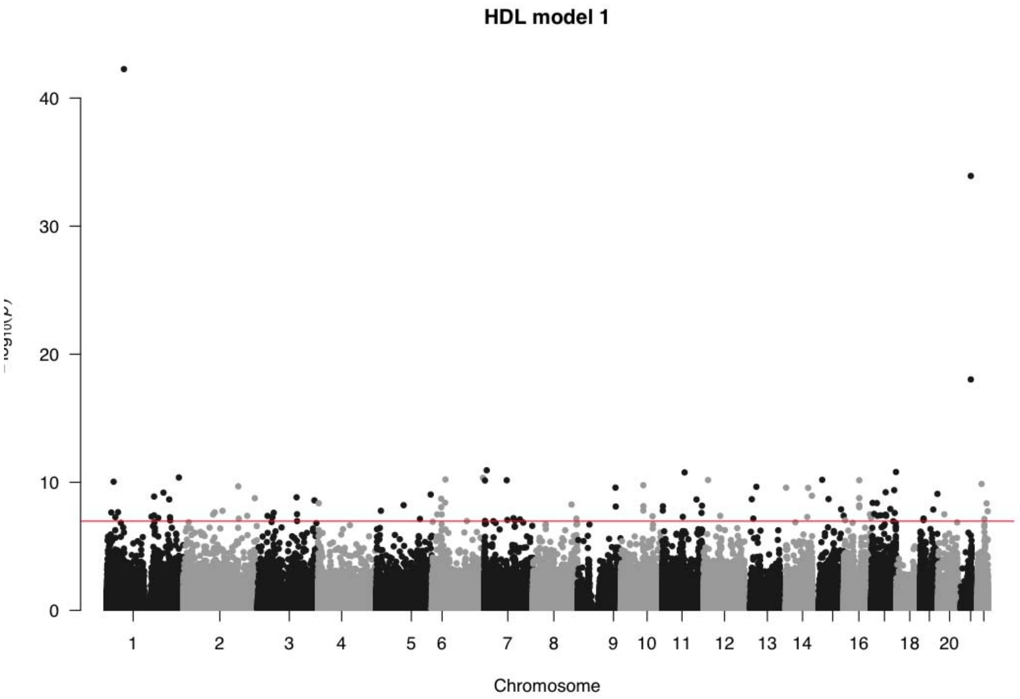

Supplementary Fig. 7. Manhattan plot of genome-wide analysis of HDL-C (model 1) in the discovery. The blue line indicates the methylome-wide significance level (*P*<108E-07).

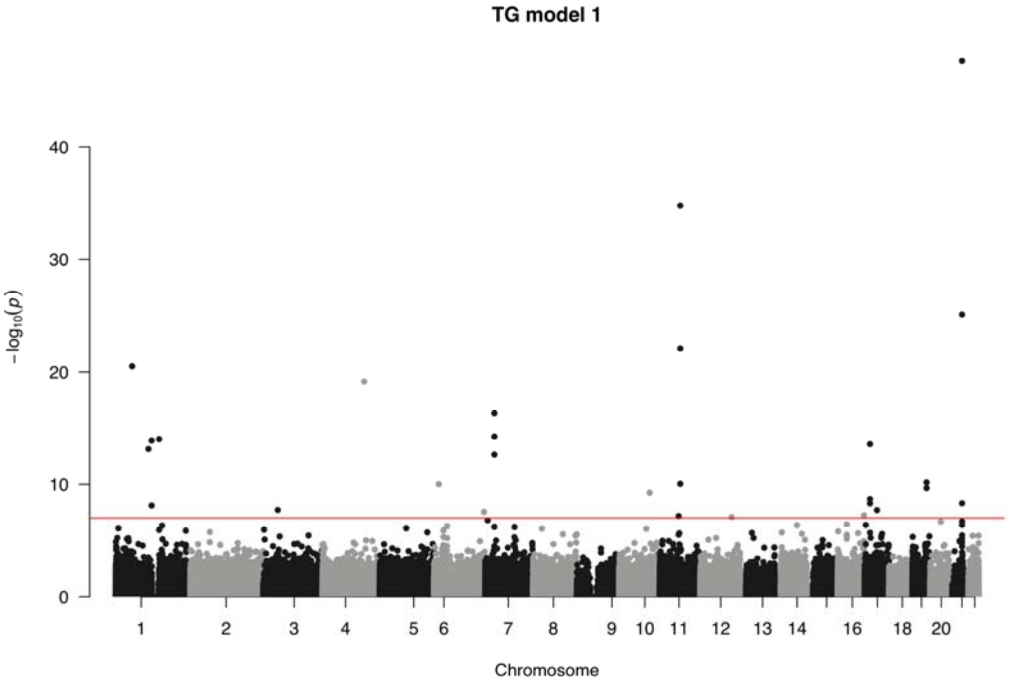

Supplementary Fig. 8. Manhattan plot of genome-wide analysis of TG (model 1) in the discovery. The red line indicates the methylome-wide significance level (*P*<108E-07).

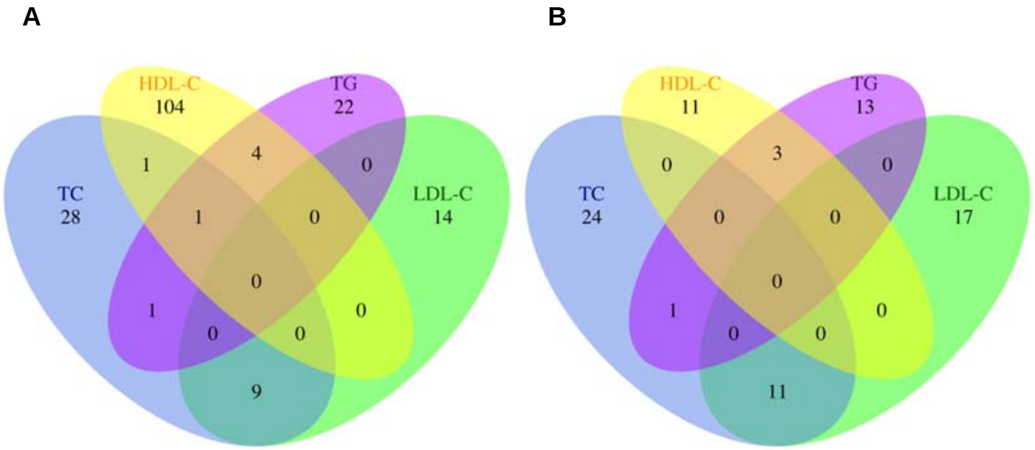

Supplementary Fig. 9. Overlap of DNA methylation sites associated with the blood lipid levels in genome-wide analyses in the discovery. A: Primary model, **B**: Secondary BMI-adjusted model.

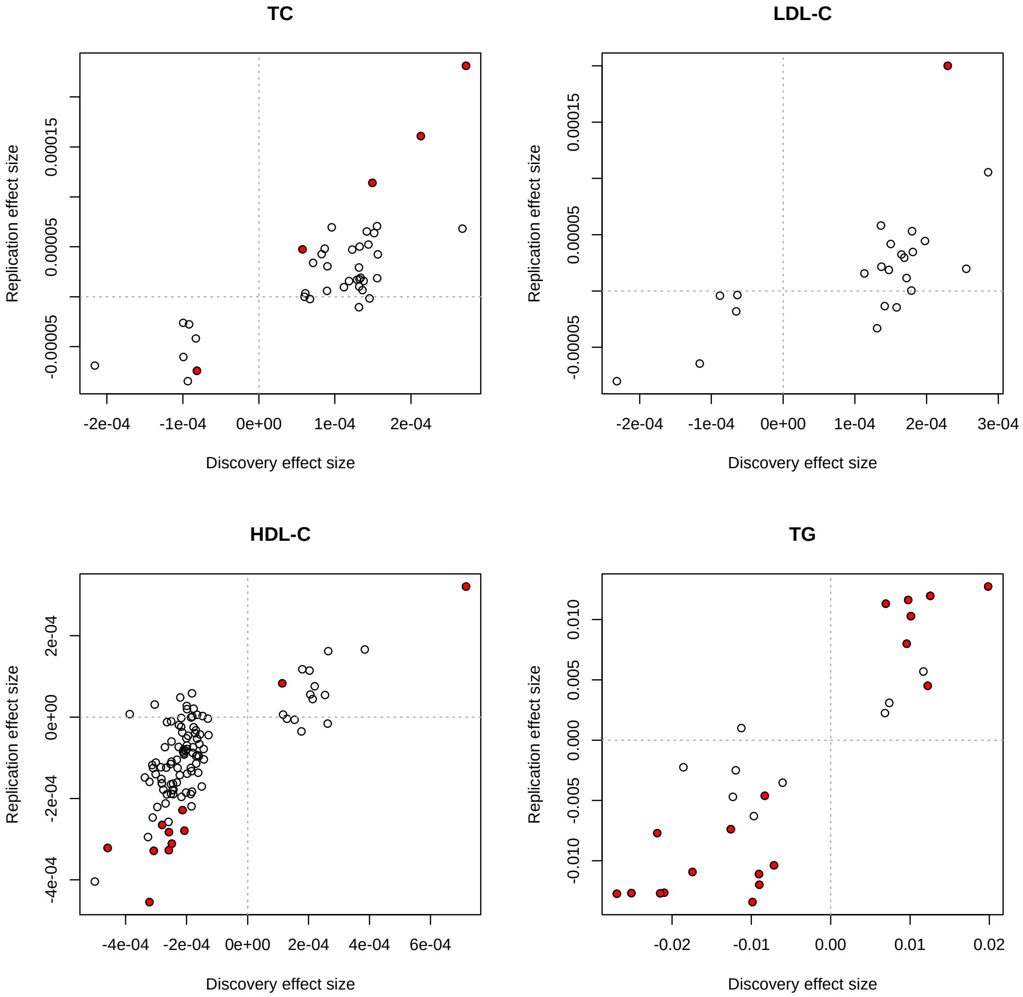

Supplementary Fig. 10. **Comparison of effect size estimates between the discovery and replication.** Plot of discovery stage coefficients versus replication stage coefficients for all CpGs significantly associated (per trait) in models 1 in the discovery. **A**. TC, Pearson correlation coefficient (*r*) = 0.78 (*r* = 0.81 following removal of replicating sites), **B**. LDL-C, *r* = 0.67 (*r* = 0.77 following removal of replicating sites), **C**. HDL-C, *r* = 0.71 (*r* = 0.70 following removal of replicating sites), and **D**. TG, *r* = 0.88 (*r* = 0.79 following removal of replicating sites). CpG sites significant also in the replication are indicated in red.

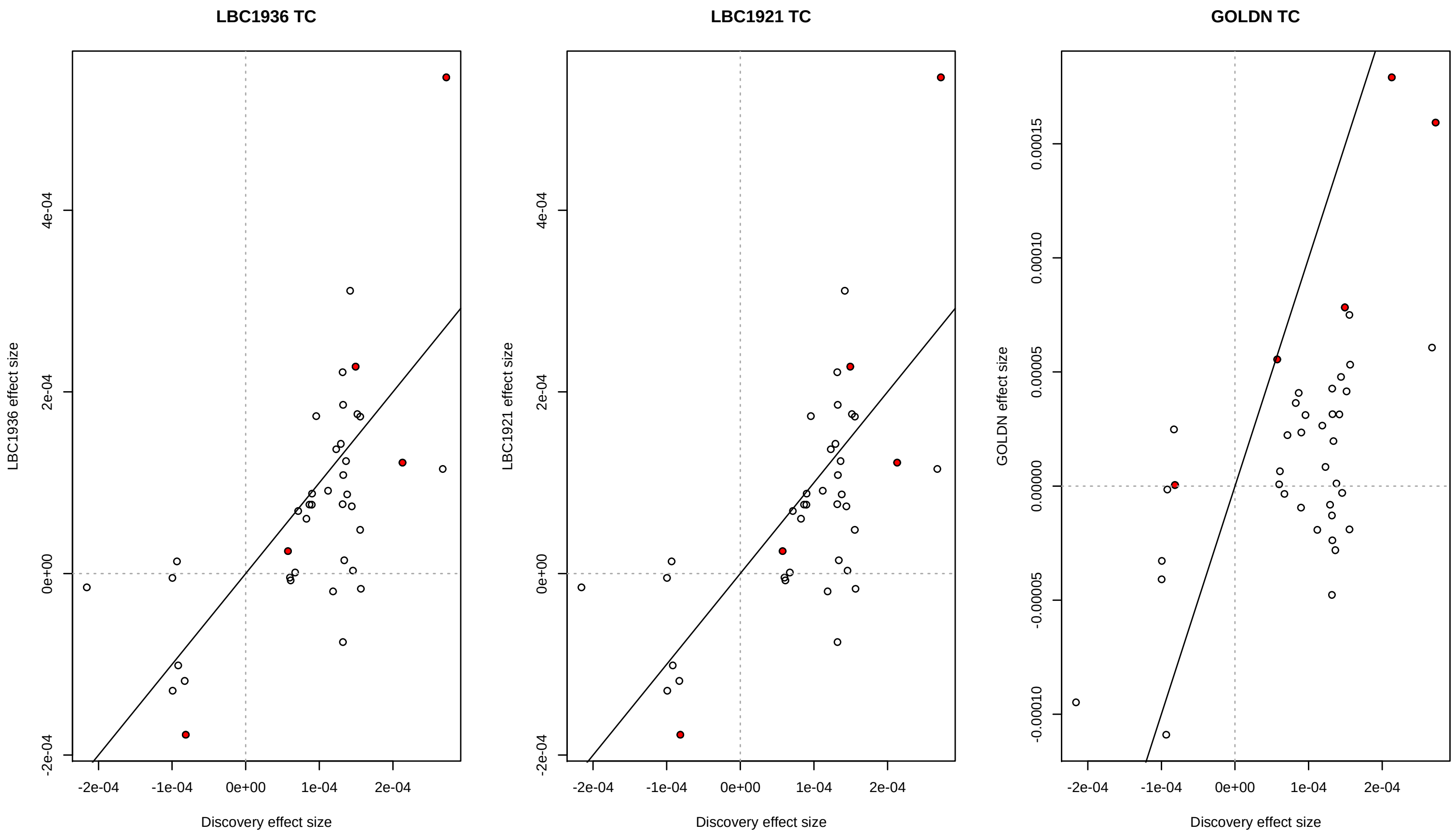

Supplementary Figure 11. Comparison of effect size estimates for TC model 1 between discovery and each of the individual replication cohorts. Beta coefficients in figure from lipid EWAS results in Discovery vs. LBC1936 (left panel), Discovery vs. LBC1921 (middle) and Discovery vs. GOLDN (right). CpG sites significant also in the replication are indicated in red. The black line represents the identity line.

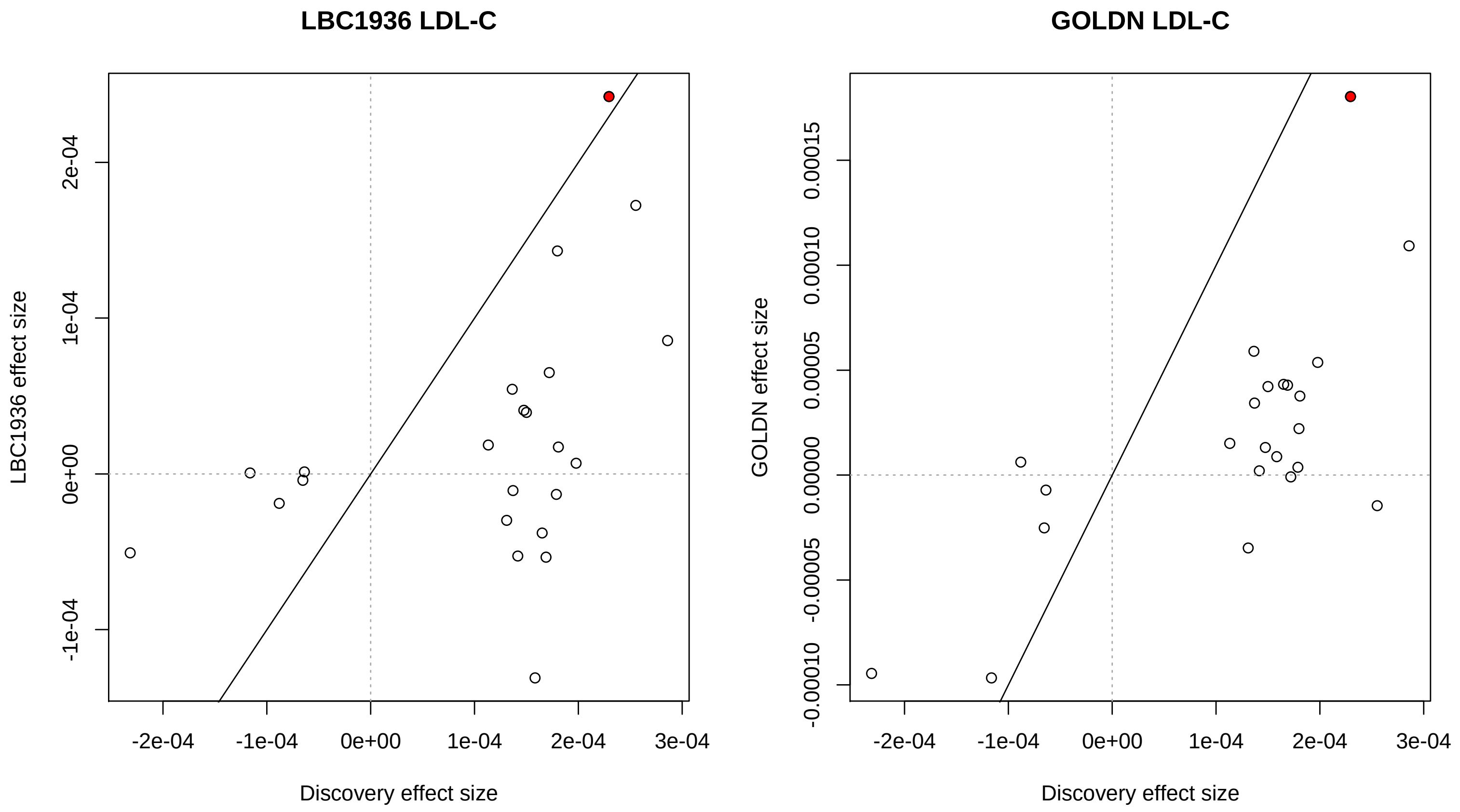

Supplementary Figure 12. Comparison of effect size estimates for LDL-C model 1 between discovery and each of the individual replication cohorts. Beta coefficients in figure from lipid EWAS results in Discovery vs. LBC1936 (left panel) and Discovery vs. GOLDN (right). CpG sites significant also in the replication are indicated in red. The black line represents the identity line.

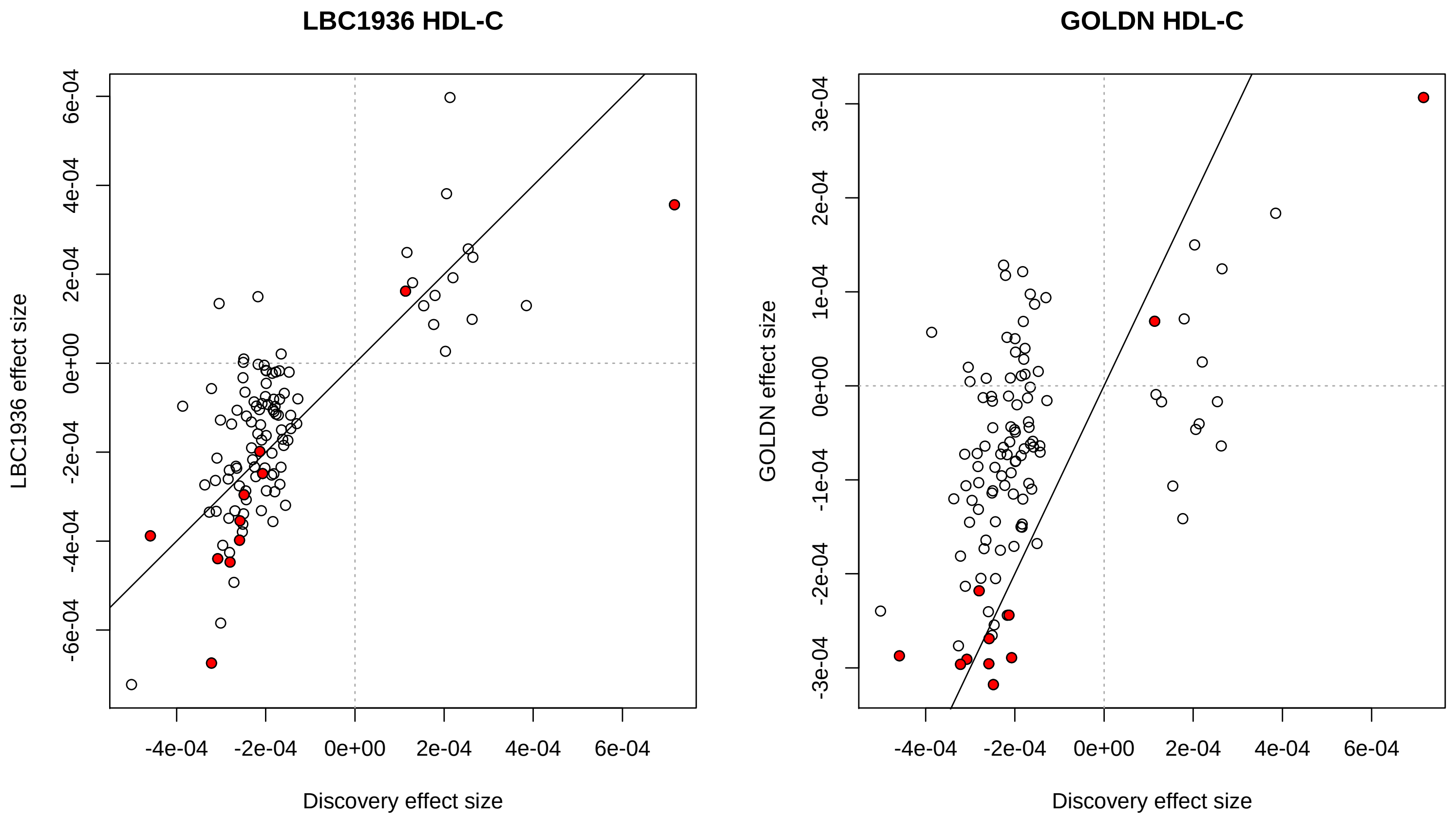

Supplementary Figure 13. Comparison of effect size estimates for HDL-C model 1 between discovery and each of the individual replication cohorts. Beta coefficients in figure from lipid EWAS results in Discovery vs. LBC1936 (left panel) and Discovery vs. GOLDN (right). CpG sites significant also in the replication are indicated in red. The black line represents the identity line.

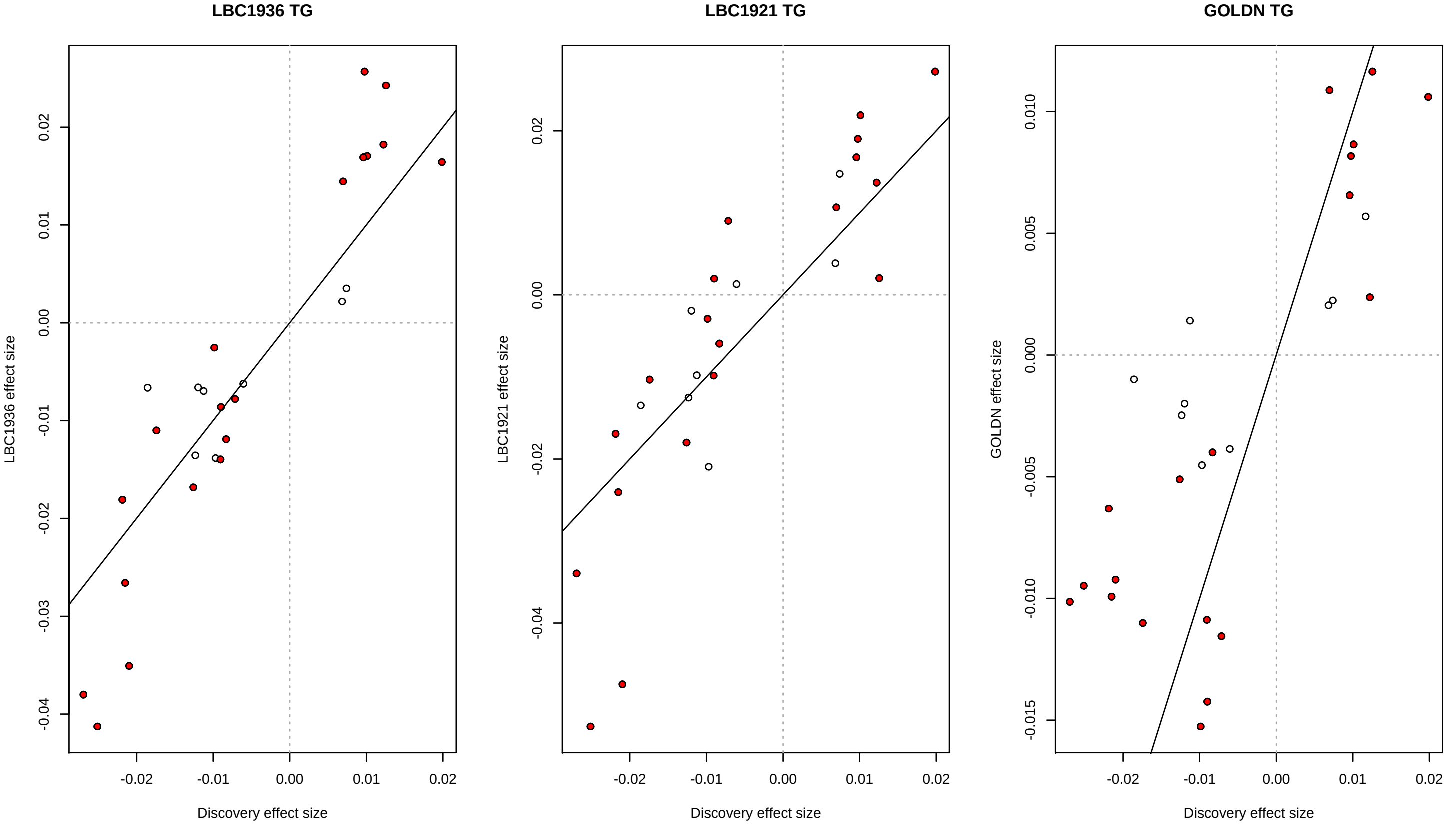

Supplementary Figure 14. Comparison of effect size estimates for TG model 1 between discovery and each of the individual replication cohorts. Beta coefficients in figure from lipid EWAS results in Discovery vs. LBC1936 (left panel), Discovery vs. LBC1921 (middle) and Discovery vs. GOLDN (right). CpG sites significant also in the replication are indicated in red. The black line represents the identity line.

A

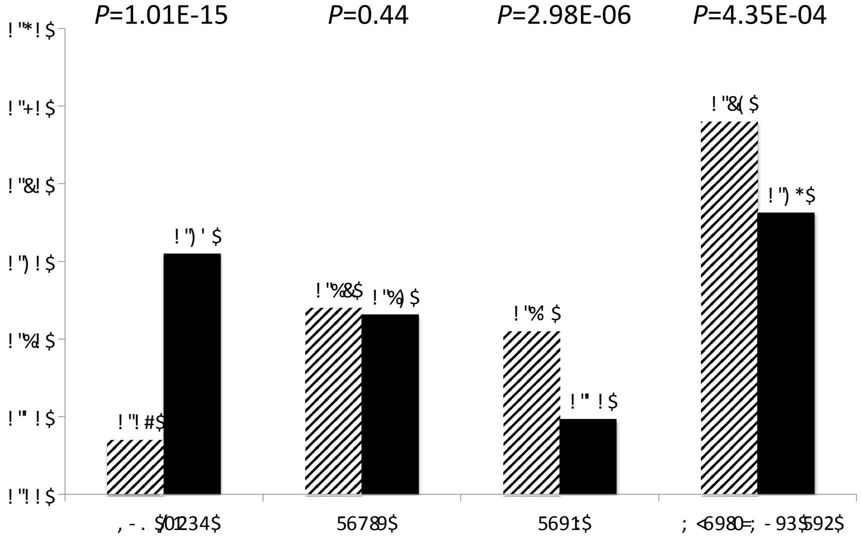

B

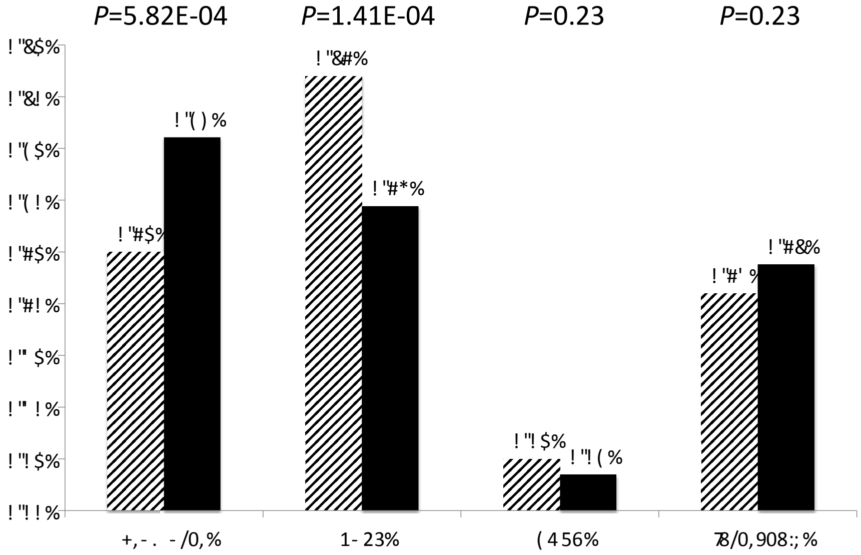

Supplementary Fig. 15. Location of lipid-associated CpGs in relation to CpG islands and genes. A. CpGs were classified into: CpG island, Shore, Shelf and Others/Open sea, and lipid-associated CpGs (striped bars) were compared with all CpGs on the array (black bars). **B.** CpGs were classified into: promoter (TSS1500, TSS200, 5'-UTR, First exon), Body, 3'-UTR and intergenic and lipid-associated CpGs (striped bars) were compared with all CpGs on the array (black bars). *Y*-axis shows the proportion in each category. P-values in figure are from a one-sided Fisher exact tests.

A

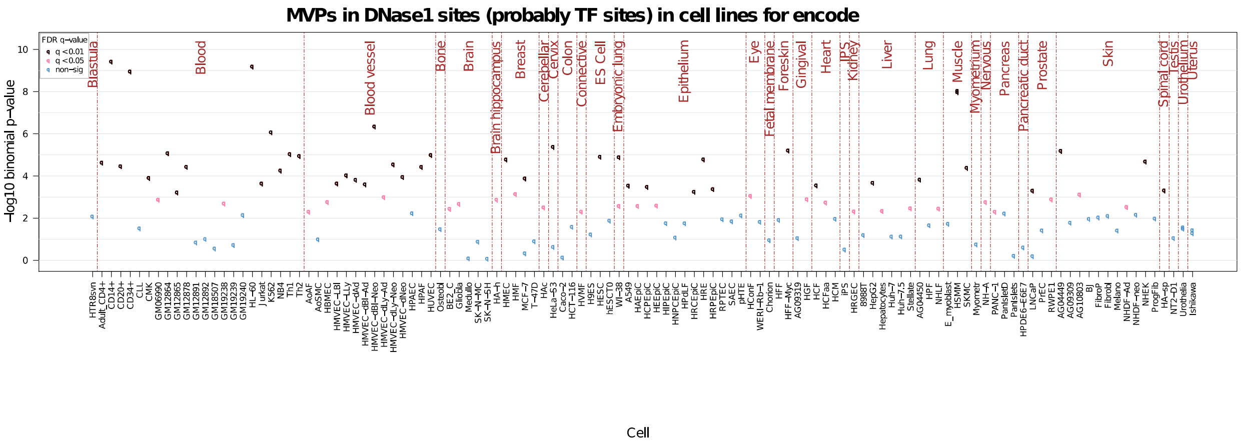

B

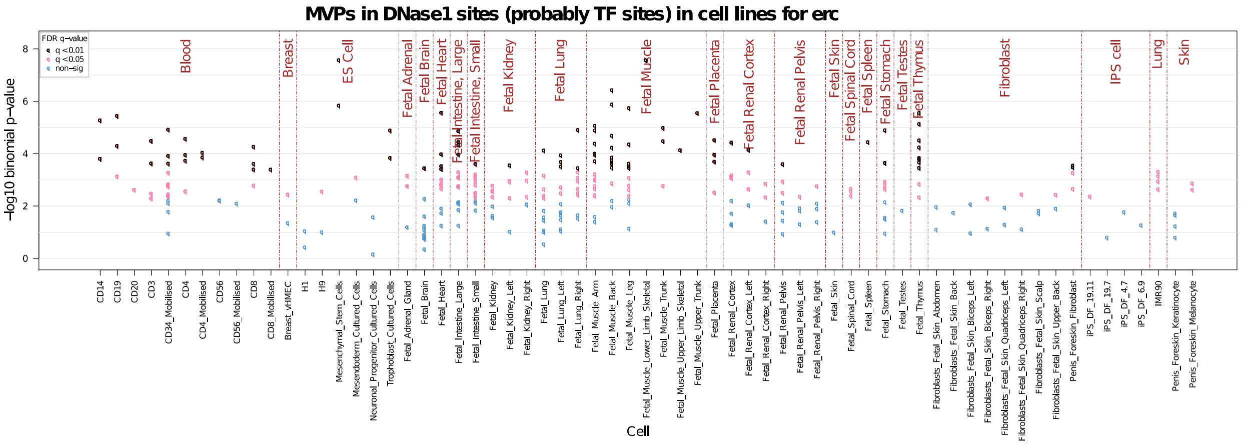

Supplementary Fig. 16. Enrichment of lipid-associated CpGs in DHS hotspots from specific tissues using eFORGE tool. A. ENCODE **B.** Roadmap Epigenome. In figure points in dark red indicate cell types with enrichment p-value of FDR q-value < 0.01.

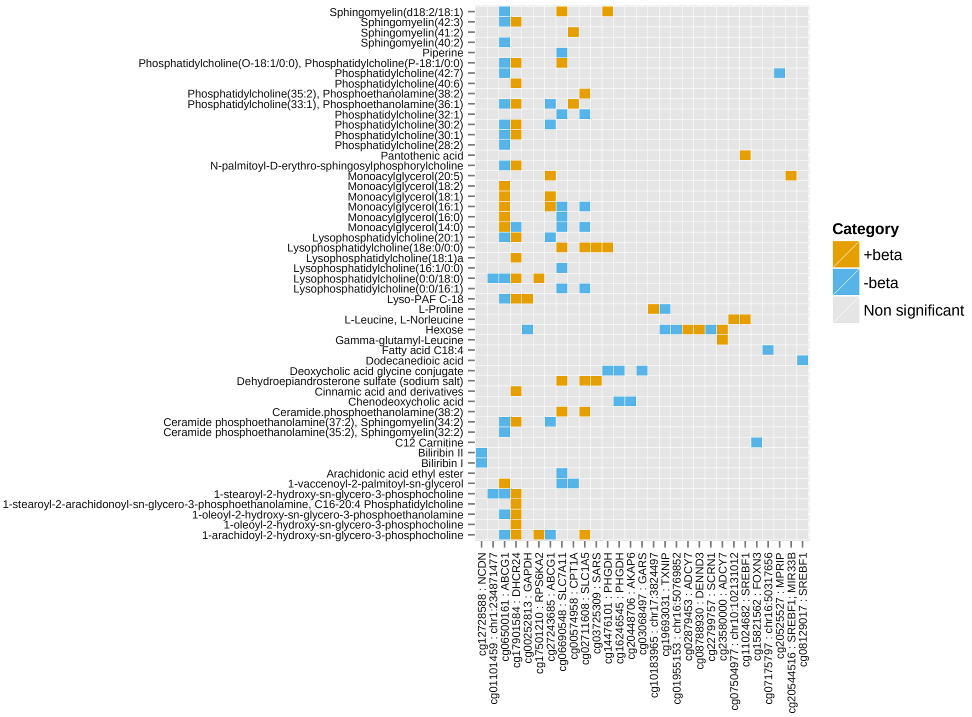

Supplementary Fig. 17. Overview of significant associations between lipid-associated CpGs and serum metabolites. Significant associations (FDR < 0.01) are depicted in orange for positively associated effects and blue for negatively associated effects. CpGs are depicted on the X-axis and metabolites on the Y-axis.

Supplementary Table 1. Cohort characteristics

	FHS	PIVUS	LBC1936	LBC1921	GOLDN
**Sample type**	Blood	Blood	Blood	Blood	T-cells
**Fasting status**	Fasted	Fasted	Non-fasted	Non-fasted	Fasted
**Age, mean (SD)**	66.4 (8.9)	70.2 (0.2)	69.5 (0.8)	79.1 (0.6)	48.8 (16)
**BMI (kg/m^2)**	28.3 (5.3)	26.9 (4.3)	27.4 (4.2)	26.1 (4.0)	28.3 (6)
**Female (%)**	54.3	49.8	52.8	60.3	52.3
**TC (mg/dL)**					
N	1494	812	654	380	991
Mean (SD)	201 (33)	215.4 (36.9)	224.9 (41.6)	221.7 (43.6)	190.0 (38)
Range	85 - 328	116 - 363.5	123.7 - 417.6	127.6 - 379.0	98- 332
**LDL (mg/dL)**					
N	1494	810	588	--	991
Mean (SD)	118 (28)	136 (31.9)	135.5 (36.7)	--	121.6 (31)
Range	26-215	42.5-266.8	33.9 - 317.6	--	44 - 236
**HDL (mg/dL)**					
N	1494	812	592	--	991
Mean (SD)	61 (19)	59.4 (16.7)	60.4 (17.4)	--	47.0 (13)
Range	22-156	23.2-146.9	26.3 - 147.7	--	22- 110
**TG (mg/dL)**					
N	1494	812	588	376	991
Mean (SD)	112 (65)	109.0 (48.4)	140.8 (64.6)	164.7 (76.9)	137.0 (95)
Range	30-790	11.5-372.0	41.6 - 419.8	62.0 - 611.1	23 - 1085

Supplementary Table 13. Genes annotated to lipid CpGs in GWAS loci of cardiovascular traits

Associated lipid trait in epigenetic analysis	Gene annotated to lipid-associated CpG(s)	GWAS trait(s)	Reference
TC, LDL-C	*APOB*	Lipids (TC, LDL-C), Coronary artery disease	[1], [2]
HDL-C	*TRIB1*	Lipids (TC, LDL-C, HDL-C, TG), Adiponectin	[1], [3]
HDL-C	*LDLR*	Lipids (TC, LDL-C), Coronary artery disease	[1], [2]
TG	*MYLIP*	Lipids (TC, LDL-C)	[1]
LDL-C	*GALNT2*	Lipids (HDL-C, TG)	[1]
HDL-C	*AMPD3*	Lipids (HDL-C)	[1]
TC, LDL-C	*FADS2*	Lipids (TC, LDL-C, HDL-C, TG)	[1]
HDL-C	*KLF13*	Waist-hip-ratio	[4]
HDL-C	*CBX3*	Waist-hip-ratio	[4]
TG	*LY86*	Waist-hip-ratio	[4]
TC	*ADCY3*	BMI	[5]
TC	*PTPRD*	Type-2 Diabetes	[6]

Supplementary Table 14. Enriched biological categories among genes annotated to TC-associated CpGs (FDR <0.05)

Category	Term	Fold	FDR
**Discovery**			
GO BP	GO:0016125~ sterol metabolic process	30.44	2.90E-03
GO BP	GO:0008203~ cholesterol metabolic process	33.42	4.00E-03
KEGG Pathway	hsa00100: Steroid biosynthesis	74.78	1.19E-02
GO BP	GO:0008202~ steroid metabolic process	15.22	2.85E-02
Panther BP	BP00019: Lipid, fatty acid and steroid metabolism	5.71	3.66E-02
**Replication**			
KEGG Pathway	hsa00100:Steroid biosynthesis	299.12	3.34E-03
UP_SEQ_FEATURE	nucleotide phosphate-binding region:FAD	313.33	2.21E-02
REACTOME Pathway	REACT_602:Metabolism of lipids and lipoproteins	22.65	4.41E-02
**mRNA expression of gene associated with CpG**			
KEGG Pathway	Antigen processing and presentation	30.63	3.76E-02
GO BP	Sterol metabolic process	57.40	4.72E-02

BP, Biological Process

Supplementary Table 15. Enriched biological categories among genes annotated to HDL-C associated CpGs (FDR <0.05)

Category	Term	Fold	FDR
**Discovery**			
N.S.	N.S.	N.S.	N.S.
**Replication**			
REACTOME Pathway	REACT_602:Metabolism of lipids and lipoproteins	22.65	4.41E-02
**mRNA expression of gene associated with CpG**			
REACTOME Pathway	REACT_602:Metabolism of lipids and lipoproteins	16.99	5.64E-03

Supplementary Table 16. Enriched biological categories among genes annotated to TG-associated CpGs (FDR <0.05)

Category	Term	Fold	FDR
**Discovery**			
Panther BP	BP00013: Amino acid metabolism	18.110	3.40E-02
**Replication**			
Panther BP	BP00013: Amino acid metabolism	26.16	5.58E-03
**mRNA expression of gene associated with CpG**			
Panther BP	BP00013: Amino acid metabolism	26.16	6.09E-03

BP, Biological Process

Supplementary Table 18. Lipid-associated CpGs significantly associated with levels of expression of neighbouring genes in blood in FHS (FDR <0.05).

CpG	CGI info	Gene Property	Gene annotated to CpG	Expression of Gene Associated with CpG	Beta	*P*	meQTL SNP is also an eQTL for associated expressed gene (*P*<1E-4)	meQTL peak SNP	eQTL peak SNP	r^2 meQTL SNP - eQTL peak SNP
cg17901584	S_Shore	TSS1500	*DHCR24*	*DHCR24*	-1.22	4.84E-13	Yes	rs6687489	rs12131972	0.72
cg19273683		Body	*ECE1*	*ECE1*	-0.73	6.75E-08	Yes	rs3026815	rs1067221	0.264
cg01751802	S_Shore	TSS1500	*KANK2*	*KANK2*	-0.41	6.50E-05	Yes	19:11317508:TG_T	rs7254270	0.001
cg26313301	S_Shelf	Body	*LDLR*	*LDLR*	0.68	8.95E-05	Yes	rs2569550	rs77373181	--
cg14476101	S_Shore	Body	*PHGDH*	*PHGDH*	-0.38	2.76E-08	Yes	rs11583993	rs11583993	Identical
cg16246545	S_Shore	Body	*PHGDH*	*PHGDH*	-0.41	3.06E-06	Yes	rs11583993	rs11583993	Identical
cg25739016		Body	*RCSD1*	*LOC100128751*	2.1	2.34E-09	Yes	rs1229359	rs7513712	0.007
cg22304262	N_Shelf	5UTR/Body	*SLC1A5*	*PRKD2*	-0.42	6.91E-07	Yes	rs8105903	rs60652743	0.15
cg08129017	S_Shore	Body	*SREBF1*	*SREBF1*	-0.51	1.07E-11	Yes	rs9899634	rs8078756	1
cg09978077	Island	Body	*SREBF2*	*SREBF2*	-2.05	9.37E-12	Yes	rs9607850	rs9611674	0.87
cg05295703		--	--	*IL1RL1*	-3.26	1.42E-18	Yes	rs12469892	rs1420103	0.734
cg05295703		--	--	*IL18R1*	-1.74	2.86E-06	Yes	rs12469892	rs10490202	0.148
cg24002003		--	--	*CHSY1*	0.88	9.63E-08	Yes	rs3784526	rs3784526	Identical
cg01176028	N_Shore	Body	*ABCG1*	*ABCG1*	-0.66	1.36E-06	No	rs225448	rs9976024	0.17
cg06500161	S_Shore	Body	*ABCG1*	*ABCG1*	-1.75	8.58E-49	No	rs225443	rs9976024	0.03
cg27243685	S_Shelf	5UTR/Body	*ABCG1*	*ABCG1*	-1.94	7.23E-28	No	rs4148086	rs9976024	0.63
cg22488164	N_Shelf	Body	*PLBD1*	*PLBD1*	-0.54	4.84E-05	No	rs2098542	rs151001109	--
cg17501210		Body	*RPS6KA2*	*RNASET2*	-0.62	9.75E-10	No	rs7745806	rs429083	*>500kb*
cg22304262	N_Shelf	5UTR/Body	*SLC1A5*	*SLC1A5*	-0.64	3.63E-07	No	rs8105903	rs3027953	0.27
cg11001536		--	--	*FAM114A2*	-0.62	2.36E-05	N.A.[1]	--	--	--
cg00574958	N_Shore	5UTR	*CPT1A*	*CPT1A*	-4.02	5.53E-19	N.A.[1]	--	--	--
cg17058475	N_Shore	5UTR	*CPT1A*	*CPT1A*	-2.47	2.20E-10	N.A.[1]	--	--	--
cg09737197	N_Shore	5UTR	*CPT1A*	*CPT1A*	-1.58	1.36E-08	N.A.[1]	--	--	--
cg08788930		Body	*DENND3*	*SLC45A4*	-1.12	1.20E-05	N.A.[1]	--	--	--
cg21645268	N_Shelf	Body	*FDFT1*	*CTSB*	-3.55	1.58E-33	N.A.[1]	--	--	--
cg21645268	N_Shelf	Body	*FDFT1*	*CTSB*	1.31	1.57E-06	N.A.[1]	--	--	--
cg18520125		Body	*FLT1*	*FLT1*	-0.69	8.94E-07	N.A.[1]	--	--	--
cg16609995		3UTR; TSS1500	*GPSM3; PBX2*	*HLA-DRB6*	-3.09	1.92E-06	N.A.[1]	--	--	--
cg16609995		3UTR; TSS1500	*GPSM3; PBX2*	*AGER; RNF5*	-0.93	3.12E-06	N.A.[1]	--	--	--
cg16609995		3UTR; TSS1500	*GPSM3; PBX2*	*SLC44A4*	-0.67	2.04E-05	N.A.[1]	--	--	--
cg09676013		ncRNA	*HLA-DPB2*	*HLA-DPB1*	-1.08	2.77E-13	N.A.[1]	--	--	--
cg09676013		ncRNA	*HLA-DPB2*	*HLA-DPA1*	-0.64	5.38E-08	N.A.[1]	--	--	--
cg09676013		ncRNA	*HLA-DPB2*	*HLA-DPB2*	0.78	3.81E-05	N.A.[1]	--	--	--
cg03717755		Body	*MYLIP*	*MYLIP*	-0.46	1.90E-05	N.A.[1]	--	--	--
cg06690548		Body	*SLC7A11*	*SLC7A11; SLC7A11-AS1*	-0.74	3.38E-15	N.A.[1]	--	--	--
cg00285394	S_Shore	Body	*SQLE*	*SQLE*	-1.42	8.28E-13	N.A.[1]	--	--	--

1. No significant meQTL

Supplementary Table 20. Association of 33 replicating lipid-associated CpGs with incident CHD[1] up to ten years after baseline in FHS and PIVUS.

			**Meta-analysis**			**FHS**		**PIVUS**			
**CpG**	**Gene**	**Gene Property**	**HR (95% CI)**[2]	***P***	**Heterogeneity *P***	**HR (95% CI)**[2]	***P***	**HR (95% CI)**[2]	***P***	**Trait(s)**[3]	**Direction CpG - lipid**
cg27243685[1]	*ABCG1*	5UTR/Body	1.38 (1.15-1.66)	6.86E-04	0.56	1.41 (1.12-1.77)	3.40E-03	1.33 (0.96-1.84)	0.079	TG (m1&m2)	+

1. 115 events in FHS and 64 events in PIVUS

2 Hazard ratio per SD increment in methylation at CpG site.

3. m1: primary model, m2: secondary BMI-adjusted model

Supplementary Methods

Cohort descriptions and sample collections

Discovery cohorts

FHS Offspring Cohort was initially recruited in 1971 and included 5,124 offspring (and their spouses) from the FHS Original Cohort [7]. In the FHS, the eligible sample for this investigation was from the 3,021 participants in the FHS Offspring Cohort who attended the eighth examination cycle in 2005-2008. The included sample was determined based on the number of participants consenting to genomic studies with available DNA and methylation assays passing quality control measures. The DNA methylation, anthropometric, and laboratory measures were obtained from the same examination. At each study visit, participants underwent a routine physical examination and a medical history interview. Participants were asked to bring in their current medication containers. Weight was measured to the nearest pound with the participant wearing only a gown without slippers or shoes, standing in the middle of the scale (Detecto Scale, Worchester Scale) with weight equally distributed on both feet. Standing height was measured to the nearest ¼ inch, with the participant barefoot or wearing thin socks, using a vertical mounted stadiometer. BMI was calculated as weight in kg divided by height in meters squared. Peripheral blood samples were collected in the morning from participants after an eight-hour fast. TC, HDL-C and TG were measured via an enzymatic colorimetric assay (Roche Hitachi 911, Roche Diagnostics) and LDL-C was calculated by the Friedewald equation. CHD was defined as a fatal or non-fatal myocardial infarction (MI), coronary death, revascularization procedure (percutaneous transluminal coronary angioplasty or coronary artery bypass graft) or coronary insufficiency (unstable angina). All CHD events were reviewed and adjudicated by a physician endpoint committee.

PIVUS is a prospective community-based cohort of participants from Uppsala, Sweden. All men and women at age 70 living in Uppsala in 2001 were invited to participate. The 1,016 participants (50% women) have been extensively phenotyped, as described previously [8], and on the Internet (www.medsci.uu.se/pivus/). The eligible sample for investigation was from the 1,016 enrolled patients at 70 years of age and conducted between the years of 2001-2003. Lipid traits were measured at Uppsala University Hospital using routine medical chemistry methods. LDL-C was calculated by the Friedewald equation. The participants have been re-examined at ages 75 and 80, and their morbidity and mortality has been followed via national registers and journal review. Clinical diagnoses by journal review of CVD up to 10 years after baseline were used to define disease events. In the present study, we combined acute fatal or non-fatal MI and revascularization procedure (percutaneous transluminal coronary angioplasty or coronary artery bypass graft) into a composite atherosclerotic CHD endpoint.

Replication cohorts

The LBC 1921 and 1936 are two longitudinal studies of ageing [9-11]. They derive from the Scottish Mental Surveys of 1932 and 1947, respectively, when nearly all 11-year old children in Scotland completed a test of general cognitive ability [9]. Survivors living in the Lothian area of Scotland were recruited in late-life at mean age 79 for LBC1921 (n=550) and mean age 70 for LBC1936 (n=1,091). Follow-up has taken place at ages 70, 73, and 76 in LBC1936 and ages 79, 83, 87, and 90 in LBC1921. The eligible sample for investigation was collected between at age 70 for LBC1936 and age 79 for LBC1921. Collected data include genetic information, longitudinal epigenetic information, longitudinal brain imaging (LBC1936), and numerous blood biomarkers, anthropomorphic and lifestyle measures. Serum cholesterol was measured as part of a blood analysis profile. Non-fasting blood was drawn on the day of cognitive assessment and analysed within 24 h in serum stored at 4 °C using an enzymatic Quinoneimine dye method measuring at 500 nm, at the Western General Hospital, Edinburgh. For LBC1921 HDL-C and LDL-C were not available.

GOLDN: The National Heart, Lung, and Blood Institute GOLDN study was designed to identify genetic determinants of lipid response to two interventions (a high-fat meal challenge and fenofibrate treatment for 3 weeks). The GOLDN study has been previously described in detail in Irvin *et al* [12]. Briefly, the study ascertained and recruited families from the Family Heart Study at two centres, Minneapolis, MN and Salt Lake City, UT, who self-reported to be white. Only families with at least two siblings were recruited for a total of 1,327 individuals. Volunteers were required to withhold lipid-lowering agents (pharmaceuticals or nutraceuticals) for at least 4 weeks prior to the initial visit to be eligible. A total of 1,053 met all eligibility requirements. The study protocol was approved by Institutional Review Boards at the University of Minnesota, University of Utah, and Tufts University/New England Medical Center. For the current study, we evaluated fasting blood lipids among 991 participants for whom baseline epigenetic data were available. Lipids were measured before the diet and drug intervention. Participants were asked to fast for ≥12 hours and abstain from alcohol intake for ≥24 hours. TG was measured by a glycerol-blanked enzymatic method (Trig/GB, Roche Diagnostics Corporation, Indianapolis, IN). TC was measured using a cholesterol esterase–cholesterol oxidase reaction (Chol R1, Roche Diagnostics Corporation) on the Roche/Hitachi 911 Automatic Analyzer (Roche Diagnostics Corporation). The same reaction was also used to measure HDL-C after precipitation of non-HDL-C with magnesium/dextran. LDL-C was measured by a homogeneous direct method (LDL Direct Liquid Select™ Cholesterol Reagent, Equal Diagnostics, Exton, PA). Data on medical history, physical activity and other lifestyle factors such as alcohol intake, smoking status, and diet were collected using an interviewer-administered questionnaire. Weight was measured by a beam balance and height was ascertained by a stadiometer. BMI was calculated as weight in kilograms divided by height in meters squared.

Genome-wide DNA methylation profiling

FHS: Buffy coat preparations were obtained from the whole blood samples and genomic DNA was extracted using the Gentra Puregene DNA extraction kit (Qiagen, Venlo, Netherlands). DNA samples were bisulphite converted using the EZ DNA Methylation kit (Zymo Research, Irvine, CA) and analysed on Illumina HumanMethylation450 chips (Illumina Inc., San Diego, CA, USA) following the manufactures' protocol. DNA methylation arrays were run in two laboratory batches at the John's Hopkins Center for Inherited Disease Research (lab batch #1) and University of Minnesota Biomedical Genomics Center (lab batch #2). The first batch included 576 samples from an earlier CVD case-control study [13] and the second batch included 2,270 samples from the remainder of the Offspring cohort participants. DNA methylation data were normalised within laboratory batches using the DASEN methodology implemented in the *wateRmelon* package [14] in R (version 3.0.2), which includes background adjustment of the methylated and unmethylated intensities and quantile normalisation of the methylated and unmethylated probes within the two types of probe technologies separately. Samples with a missing rate >1% at $P<0.01$ (n=10 for batch #1 and n=35 for batch #2), poor single nucleotide polymorphism (SNP) matching to the 65 SNP control probe locations (n=38 for batch #1 and n=41 for batch #2), and outliers by multi-dimensional scaling techniques (n=25 for batch #1 and n=48 for batch #2) were excluded. Probes with missing rate >20% at $P<0.01$ (n=466 from batch #1 and n=366 from batch #2), as well as probes previously identified to map to multiple locations [15] or to have an underlying SNP (minor allele frequency [MAF] >5% in European ancestry (EUR) 1000 genomes project data) at the CpG site or within 10 bp of the single base extension (n=42,251) were excluded. Following quality control, DNA methylation data from 2,377 FHS participants and 443,252 probes remained for analyses. The FHS methylation data are available at dbGaP under the accession number phs000724.v2.p9.

PIVUS: Blood for DNA methylation assay were collected at baseline (at age 70). Genomic DNA was extracted from blood samples and bisulphite conversion of 500ng genomic DNA was performed using the EZ-96 DNA Methylation Gold Kit (Zymo Research Product). The equivalent of approximately 200ng of bisulphite converted DNA, was removed, evaporated to a volume of <4μl, and used for methylation profiling using the Illumina Infinium assay and

the Illumina HumanMethylation450 v.1.2 bead chip according to the protocol from the supplier (Illumina Inc., San Diego, CA, USA). The results were analysed with GenomeStudio 2011.1 (Illumina Inc., San Diego, CA, USA). After exclusion of replicates a total of 1,002 study participants had methylation data available for quality control procedures. Three samples were excluded based on poor bisulphite conversion efficiency, twelve samples due to low pass rate of CpG sites (<98.5% with a detection P>0.01) and a further six samples based on low SNP genotype match (>1 SNP mismatches) between genotypes from the methylation array and Omni/Metabochip genotyping chips leaving 981 individuals with adequate methylation data available for analyses. Following removal of participants with abnormal leukocyte cell counts (>10x10^9 cells/L; n=14) methylation data from 967 individuals remained for analyses. The signal intensities for the methylated and unmethylated state were then quantile normalised for each probe type separately, and beta values were calculated.
LBC 1921 and 1936: Detailed information about the collection and QC steps undertaken on the LBC methylation data has been reported previously [16]. Briefly, the Infinium HumanMethylation450 BeadChip (Illumina Inc, San Diego, CA) was used to measure DNA methylation in whole blood of consenting participants. Background correction was performed using the R *minfi* package [17] and QC was used to remove probes with a low detection rate (<95% detection rate at P<0.01), probes with low quality (manual inspection), samples with a low call rate (samples with <450,000 probes detected at P<0.01), and samples with a poor match between genotypes and SNP control probes (cross-checked using *wateRmelon* package [14]) or incorrect predicted sex. Post QC, DNA methylation data were available for 446 LBC1921 participants at age 79 and for 920 LBC1936 participants at age. The LBC methylation data are available at European Genome-Phenome Archive under accession number EGAS00001000910.
GOLDN: DNA was extracted from CD4+ T-cells harvested from stored buffy coats using antibody-linked Invitrogen Dynabeads [18]. Stored buffy coats were collected at the same time lipid concentrations were measured. We lysed cells captured on the beads and extracted DNA using DNeasy kits (Qiagen, Venlo, Netherlands) and methylation was assayed across ~470,00 autosomal CpG sites using the Illumina Infinium Human Methylation450 Beadchip (Illumina, San Diego, CA). For each assay, 500ng of DNA was treated with sodium bisulfite (EZ DNA, Zymo Research, Irvine, CA) prior to standard Illumina amplification, hybridization, and imaging steps. The resulting intensity files were analyzed with Illumina's GenomeStudio which generated beta scores (i.e. the proportion of total signal from the methylation specific probe or color channel) and "detection P-values" (the probability that the total intensity for a given probe falls within the background signal intensity). Beta scores with an associated detection P-value greater than 0.01 were removed and samples with more than 1.5% missing data points were eliminated from further analysis. Furthermore, any CpG probes where more than 10% of samples failed to yield adequate intensity were removed. A total of 58 samples were removed. The filtered beta scores were then subjected to batch normalization with the ComBat package for R software in non-parametric mode [19]. We performed the normalization in parallel on random subsets of 20,000 CpGs per run where each array of 12 samples was used as a "batch." These methods have been extensively described in Absher *et al* and the utility of ComBat to correct for batch effects in comparison to other programs is reported [20, 21]. To correct for probe chemistry, we separately normalized probes from the Infinium I and II chemistries and subsequently adjusted the β scores for Infinium II probes using the equation derived from fitting a second order polynomial to the observed methylation values across all pairs of probes located <50bp apart (within-chemistry correlations >0.99), where one probe was Infinium I and one was Infinium II. Finally, we eliminated any CpGs where the probe sequence mapped either to a location that did not match the annotation file or to more than one locus. We identified such markers by re-aligning all probes (with unconverted Cs) to the human reference genome. After these quality control procedures, there were methylation data from 461,281 CpGs. Principal components (PCs) based on the beta scores of all autosomal CpGs passing QC were generated using the *prcomp* function in R (V 2.12.1) and used to adjust for cell purity in association analysis. Deconvolution estimated CD4+ T-cell percentages were calculated adapting the method of Abbas *et al* [22]. Predicted CD4+ T-cell

percent purity was highly correlated with PC1 (r^2=0.85, *P*=4E-293) but not other PCs, thus supporting the usefulness of methylation PCs in adjusting for cell purity in our analysis. The GOLDN methylation data are available at dbGaP under the accession number phs000741.v1.p1.

Association of methylation of blood cell-derived DNA with lipid levels

FHS: Linear mixed effects regression models were conducted to test the association between site-specific DNA methylation and each lipid phenotype (TC, HDL-C, LDL-C, TG) individually. Participants currently on lipid lowering medication were excluded. The primary model was adjusted for age, sex and technical covariates. The secondary model was additionally adjusted for BMI. Technical covariates included chip, row, column (specified as random effects) and two methylation principal components (PCs) to adjust for unmeasured batch effects. Cell count heterogeneity was accounted for by adjusting for imputed cell counts obtained by the Houseman method [23]. The linear mixed effect models were run with the *pedigreemm* package in R (version 3.1), which additionally accounts for the family correlation structure in the FHS. Familial relatedness was obtained by reported relationships and genetic similarity calculated by identity-by-descent (IBD) probabilities. For any inconsistencies between reported and IBD relationships, relationships obtained from IBD probabilities were utilized. After removing individuals on lipid-lowering medication and thus with missing phenotype, a total of 1,494 participants were considered in the association analyses.

PIVUS*:* The associations between normalised DNA methylation beta values and phenotypes were modelled by a linear mixed effect model, using R [24] and the *lmer* function (lme4 package), fitted by maximum-likelihood assuming a normally distributed error term. Models were adjusted for age, sex and predicted white cell counts (estimated from the DNA methylation data using the Houseman algorithm [23] as implemented in R package *minfi* for Illumina HumanMethylation450 [17]) as fixed effects and chip, chip row and chip column as random effects. A likelihood ratio test was used to assess the significance of the phenotype effect. The *p-value* of the phenotype effect in each model was calculated from the Chi-square distribution with 1 degree of freedom using -2log(likelihood ratio) as the test statistic. After removing individuals on lipid-lowering medication (n=155), a total of 812 individuals were considered in the association analyses.

LBC 1936 and 1921: Linear regression modelling was used to assess the association between DNA methylation (outcome variable) and the lipid traits (predictor variable). Covariates included age, sex, and measured white blood cell counts (eosinophils, basophils, neutrophils, monocytes, and lymphocytes). Additional adjustments were made for BMI in secondary models. Participants were excluded from the analyses if they were taking lipid-lowering medication. All statistical analyses were performed using R software (http://cran.r-project.org/). After removing individuals on lipid-lowering medication and thus with missing phenotype, a total of 654, 588, 592 and 588 participants were considered in the association analyses of TC, LDL-C, HDL-C and TG, respectively for LBC1936. For LBC1921, a total of 380 and 376 were considered in the association analyses of TC and TG, respectively.

GOLDN: Associations between normalised methylation beta values at each CpG site and lipid traits were analysed using mixed linear regression models adjusted for age, gender, study site, and 4 methylation PCs (as a proxy for cell purity) as fixed effects and family structure as a random effect using the R kinship package (*lmekin* function). A second set of models additionally adjusted for BMI. After removing four observations due to missing phenotype or covariate data, a total of 991 participants were considered in the association analysis.

Genotyping and Imputation

FHS: SNP data were obtained from the Affymetrix 550K Array (Affymetrix, Santa Clara, CA) and imputed to 1000 Genomes SNPs (phase 1 release), as previously reported [25]. The FHS genotype data are available at dbGaP under the accession number phs000342.v13.p9.

PIVUS*:* Individuals were genotyped using the Illumina OmniExpress and Illumina Metabochip microarrays. Prior to imputation, quality control was performed. Exclusion of

samples were performed based on the following criteria: genotype call rate <95%; heterozygosity >3 SD; gender discordance; duplicated samples; identity-by-descent match; and ethnic outliers. Monomorphic SNPs; or SNPs with Hardy-Weinberg equilibrium $P<1x10^{-6}$; genotype call rate<0.99 (SNPs with MAF<5%) or <0.95 (SNPs with MAF≥5%); MAF<1% were excluded from analysis. Data were imputed to 1000G (version: March 2012) using Impute v.2.2.2 [26].

Gene expression profiling
Gene expression data were available for participants in the FHS. RNA was extracted from whole blood using the PAXgene Blood RNA System Kit (Qiagen, Venlo, Netherlands) with mRNA expression profiling assessed using the Affymetrix Human Exon 1.0 ST GeneChip platform. Gene expression data were normalised using robust multichip average methods [27] with quality control measures as previously described [13]. Cell count proportions were derived from gene expression markers in this sample set as there was overlap between gene expression measures and directly measured cell counts (lymphocytes, monocytes, neutrophils, basophils, and eosinophils) from a sample of 2,280 Third Generation FHS participants obtained during the second examination cycle (2008-2011). Internal validation using training and testing datasets achieved an $r^2 > 0.8$ in the majority of cell lines (except basophils). The FHS gene expression data are available at dbGaP under the accession number phs000363.v12.p9.

Metabolomic profiling
Metabolomics data were available for participants in the PIVUS. Untargeted metabolomic profiling of serum samples was measured in duplicates as described previously [28]. In brief, 1 μl of sample was analysed on Acquity UPLC coupled to a Xevo G2 Q-TOFMS (Waters Corporation, Milford, Massachusetts, USA) and raw data was processed using XCMS software [29] for detection, alignment, grouping, and imputation of features. For normalisation of data metabolic feature intensities were log-transformed and an ANOVA-type normalisation applied. Fragmentation spectra were reconstructed from metabolic features with strong correlation and similar retention time and metabolites were identified from spectra. Only annotated metabolites (n=229) were used in analysis in relation to DNA methylation. Raw spectra from mass spectrometry analysis and annotated metabolites intensities are available in Metabolights (http://www.ebi.ac.uk/metabolights/) with accession number MTBLS90.

References

1. Willer CJ, Schmidt EM, Sengupta S, Peloso GM, Gustafsson S, Kanoni S, Ganna A, Chen J, Buchkovich ML, Mora S, et al. Discovery and refinement of loci associated with lipid levels. *Nat Genet*. 2013;45:1274-1283
2. Deloukas P, Kanoni S, Willenborg C, Farrall M, Assimes TL, Thompson JR, Ingelsson E, Saleheen D, Erdmann J, Goldstein BA, et al. Large-scale association analysis identifies new risk loci for coronary artery disease. *Nat Genet*. 2013;45:25-33
3. Dastani Z, Hivert MF, Timpson N, Perry JR, Yuan X, Scott RA, Henneman P, Heid IM, Kizer JR, Lyytikainen LP, et al. Novel loci for adiponectin levels and their influence on type 2 diabetes and metabolic traits: A multi-ethnic meta-analysis of 45,891 individuals. *PLoS Genet*. 2012;8:e1002607
4. Shungin D, Winkler TW, Croteau-Chonka DC, Ferreira T, Locke AE, Magi R, Strawbridge RJ, Pers TH, Fischer K, Justice AE, et al. New genetic loci link adipose and insulin biology to body fat distribution. *Nature*. 2015;518:187-196
5. Locke AE, Kahali B, Berndt SI, Justice AE, Pers TH, Day FR, Powell C, Vedantam S, Buchkovich ML, Yang J, et al. Genetic studies of body mass index yield new insights for obesity biology. *Nature*. 2015;518:197-206
6. Tsai FJ, Yang CF, Chen CC, Chuang LM, Lu CH, Chang CT, Wang TY, Chen RH, Shiu CF, Liu YM, et al. A genome-wide association study identifies susceptibility variants for type 2 diabetes in han chinese. *PLoS Genet*. 2010;6:e1000847

7. Kannel WB, Feinleib M, McNamara PM, Garrison RJ, Castelli WP. An investigation of coronary heart disease in families. The framingham offspring study. *Am J Epidemiol*. 1979;110:281-290
8. Lind L, Fors N, Hall J, Marttala K, Stenborg A. A comparison of three different methods to evaluate endothelium-dependent vasodilation in the elderly: The prospective investigation of the vasculature in uppsala seniors (pivus) study. *Arterioscler Thromb Vasc Biol*. 2005;25:2368-2375
9. Deary IJ, Whiteman MC, Starr JM, Whalley LJ, Fox HC. The impact of childhood intelligence on later life: Following up the scottish mental surveys of 1932 and 1947. *J Pers Soc Psychol*. 2004;86:130-147
10. Deary IJ, Gow AJ, Taylor MD, Corley J, Brett C, Wilson V, Campbell H, Whalley LJ, Visscher PM, Porteous DJ, et al. The lothian birth cohort 1936: A study to examine influences on cognitive ageing from age 11 to age 70 and beyond. *BMC Geriatr*. 2007;7:28
11. Deary IJ, Gow AJ, Pattie A, Starr JM. Cohort profile: The lothian birth cohorts of 1921 and 1936. *Int J Epidemiol*. 2012;41:1576-1584
12. Irvin MR, Kabagambe EK, Tiwari HK, Parnell LD, Straka RJ, Tsai M, Ordovas JM, Arnett DK. Apolipoprotein e polymorphisms and postprandial triglyceridemia before and after fenofibrate treatment in the genetics of lipid lowering and diet network (goldn) study. *Circ Cardiovasc Genet*. 2010;3:462-467
13. Joehanes R, Ying S, Huan T, Johnson AD, Raghavachari N, Wang R, Liu P, Woodhouse KA, Sen SK, Tanriverdi K, et al. Gene expression signatures of coronary heart disease. *Arterioscler Thromb Vasc Biol*. 2013;33:1418-1426
14. Pidsley R, CC YW, Volta M, Lunnon K, Mill J, Schalkwyk LC. A data-driven approach to preprocessing illumina 450k methylation array data. *BMC Genomics*. 2013;14:293
15. Chen YA, Lemire M, Choufani S, Butcher DT, Grafodatskaya D, Zanke BW, Gallinger S, Hudson TJ, Weksberg R. Discovery of cross-reactive probes and polymorphic cpgs in the illumina infinium humanmethylation450 microarray. *Epigenetics*. 2013;8:203-209
16. Shah S, McRae AF, Marioni RE, Harris SE, Gibson J, Henders AK, Redmond P, Cox SR, Pattie A, Corley J, et al. Genetic and environmental exposures constrain epigenetic drift over the human life course. *Genome Res*. 2014;24:1725-1733
17. Aryee MJ, Jaffe AE, Corrada-Bravo H, Ladd-Acosta C, Feinberg AP, Hansen KD, Irizarry RA. Minfi: A flexible and comprehensive bioconductor package for the analysis of infinium DNA methylation microarrays. *Bioinformatics*. 2014;30:1363-1369
18. Neurauter AA, Bonyhadi M, Lien E, Nokleby L, Ruud E, Camacho S, Aarvak T. Cell isolation and expansion using dynabeads. *Adv Biochem Eng Biotechnol*. 2007;106:41-73
19. Johnson WE, Li C, Rabinovic A. Adjusting batch effects in microarray expression data using empirical bayes methods. *Biostatistics*. 2007;8:118-127
20. Chen C, Grennan K, Badner J, Zhang D, Gershon E, Jin L, Liu C. Removing batch effects in analysis of expression microarray data: An evaluation of six batch adjustment methods. *PLoS One*. 2011;6:e17238
21. Absher DM, Li X, Waite LL, Gibson A, Roberts K, Edberg J, Chatham WW, Kimberly RP. Genome-wide DNA methylation analysis of systemic lupus erythematosus reveals persistent hypomethylation of interferon genes and compositional changes to cd4+ t-cell populations. *PLoS Genet*. 2013;9:e1003678
22. Abbas AR, Wolslegel K, Seshasayee D, Modrusan Z, Clark HF. Deconvolution of blood microarray data identifies cellular activation patterns in systemic lupus erythematosus. *PLoS One*. 2009;4:e6098
23. Houseman EA, Accomando WP, Koestler DC, Christensen BC, Marsit CJ, Nelson HH, Wiencke JK, Kelsey KT. DNA methylation arrays as surrogate measures of cell mixture distribution. *BMC Bioinformatics*. 2012;13:86

24. Team RC. *R: A language and environment for statistical computing.* Vienna, Austria: R Foundation for Statistical Computing; 2015.
25. Zhang X, Johnson AD, Hendricks AE, Hwang SJ, Tanriverdi K, Ganesh SK, Smith NL, Peyser PA, Freedman JE, O'Donnell CJ. Genetic associations with expression for genes implicated in GWAS studies for atherosclerotic cardiovascular disease and blood phenotypes. *Hum Mol Genet*. 2014;23:782-795
26. Marchini J, Howie B, Myers S, McVean G, Donnelly P. A new multipoint method for genome-wide association studies by imputation of genotypes. *Nat Genet*. 2007;39:906-913
27. Irizarry RA, Hobbs B, Collin F, Beazer-Barclay YD, Antonellis KJ, Scherf U, Speed TP. Exploration, normalization, and summaries of high density oligonucleotide array probe level data. *Biostatistics*. 2003;4:249-264
28. Ganna A, Salihovic S, Sundstrom J, Broeckling CD, Hedman AK, Magnusson PK, Pedersen NL, Larsson A, Siegbahn A, Zilmer M, et al. Large-scale metabolomic profiling identifies novel biomarkers for incident coronary heart disease. *PLoS Genet*. 2014;10:e1004801
29. Smith CA, Want EJ, O'Maille G, Abagyan R, Siuzdak G. Xcms: Processing mass spectrometry data for metabolite profiling using nonlinear peak alignment, matching, and identification. *Anal Chem*. 2006;78:779-787
